# Supplementary material for: Macrophage RIPK3 triggers inflammation and cell death via the XBP1–Foxo1 axis in liver ischaemia–reperfusion injury
Source: JHEP Rep. 2023 Aug 12;5(11):100879. doi: 10.1016/j.jhepr.2023.100879 (PMC10568422; doi:10.1016/j.jhepr.2023.100879)
Supplement: Multimedia component 1 [file mmc1.pdf]

Macrophage RIPK3 triggers inflammation and cell death via the XBP1–Foxo1 axis in liver ischaemia–reperfusion injury

Xiaoye Qu, Tao Yang, Xiao Wang, Dongwei Xu, Yeping Yu, Jun Li, Longfeng Jiang, Qiang Xia, Douglas G. Farmer, Bibo Ke

Table of contents

Supplementary materials and methods..2

Fig. S1..9

Fig. S2..10

Fig. S3..11

Fig. S4..12

Fig. S5..13

Fig. S6..14

Fig. S7..15

Table S1..16

Supplementary references...17

Supplementary materials and methods

Animals. The floxed RIPK3 ($RIPK3^{FL/FL}$) mice (B6;129-$RIPK3^{tm1.1Fkmc}$/J) and the mice expressing Cre recombinase under the control of the Lysozyme 2 (Lyz2) promoter (LysM-Cre) were obtained from The Jackson Laboratory (Bar Harbor, ME). The targeting vector is designed to insert a loxP site and a FRT-flanked neomycin resistance (neo) upstream of exon 10. An enhanced green fluorescent protein (EGFP) sequence, followed by a second loxP site, is inserted at the end of the coding region. Flp-mediated recombination removed the FRT-flanked neo cassette. This strain was maintained on a mixed 129 and C57BL/6 genetic background. To generate myeloid-specific RIPK3 knockout ($RIPK3^{M\text{-}KO}$) mice, a homozygous loxP-flanked RIPK3 mouse was mated with a homozygous Lyz2-Cre mouse to create the F1 mice that were heterozygous for a loxP-flanked RIPK3 allele and heterozygous for the Lyz2-Cre. The F1 mice were then backcrossed to the homozygous loxP-flanked RIPK3 mice, resulting in the generation of $RIPK3^{M\text{-}KO}$ (25% of the offspring), which were homozygous for the loxP-flanked RIPK3 allele and heterozygous for the Lyz2-Cre allele (Fig. S1). The myeloid-specific Foxo1 knockout ($Foxo1^{M\text{-}KO}$) mice were generated as described [1]. Mouse genotyping was performed using a standard protocol with primers described in the JAX Genotyping protocols database. Male mice at 6-8 weeks of age were used in all experiments. This study was performed in strict accordance with the recommendations in the *Guide for the Care and Use of Laboratory Animals* published by the National Institutes of Health. Animal protocols were approved by the Institutional Animal Care and Use Committee of The University of California at Los Angeles.

Mouse liver IRI model. We used an established mouse model of warm hepatic ischemia (90min) followed by reperfusion (6h) [2]. Mice were injected with heparin (100U/kg), and an atraumatic clip was used to interrupt the arterial/portal venous blood supply to the cephalad liver lobes. After 90min of ischemia, the clip was removed, and mice were sacrificed at 6h of reperfusion. Some animals were injected via tail vein with Zc3h15-expressing bone marrow-

derived macrophages (BMMs) or control cells ($1x10^6$ cells in 0.1 ml of PBS/mouse) 24h before ischemia.

Hepatocellular function assay. Serum alanine aminotransferase (sALT) levels, an indicator of hepatocellular injury, were measured by ALT and AST kit (ThermoFisher, Waltham, MA) according to the manufacturer's instructions.

Histology, immunohistochemistry, and immunofluorescence staining. Liver sections (5-μm) were stained with hematoxylin and eosin (H&E). The severity of IRI was graded using Suzuki's criteria [3]. Liver macrophages were detected using primary rat $CD11b^+$ monoclonal antibodies (mAb) (Abcam, Cambridge, MA) and secondary AlexFluor488-conjugated AffiniPure donkey anti-rat IgG (Jackson Immunoresearch, West Grove, PA) for immunofluorescence staining. DAPI was used for nuclear counterstaining. Liver neutrophils were detected by immunohistochemistry (IHC) or Immunofluorescence staining using primary rat Ly6G mAb (ThermoFisher Scientific). Immunofluorescence staining of RIPK3 in Kupffer cells or TRMP7 in hepatocytes were analyzed in the liver sections using primary rabbit RIPK3 mAb (Cell Signaling Technology, Danvers, MA) and rat CD68 mAb (Bio-Rad, Hercules, CA) or mouse TRMP7 mAb (Santa Cruz Biotechnology) and rabbit HNF4α mAb (Abcam). The gene expression was detected in liver sections by Immunofluorescence and immunohistochemistry staining using primary rabbit Zc3h15 (ThermoFisher Scientific) and mouse NOD1 (Santa Cruz Biotechnology) Abs. The primary rabbit XBP1s (Cell Signaling Technology) and mouse Foxo1 (Santa Cruz Biotechnology) Abs, the secondary AlexFluor488-conjugated AffiniPure donkey anti-rabbit IgG Ab, Cy5-conjugated AffiniPure donkey anti-mouse IgG Ab (Jackson Immunoresearch) were used for staining XBP1s and Foxo1 positive cells according to the manufacturer's instructions. Images for immunofluorescence staining were captured using a fluorescence microscope (Keyence BZ-X810, Osaka, Japan) and analyzed using Image-pro Plus software. Positive cells were counted blindly in 10 HPF/section (x200).

Quantitative RT-PCR analysis. Total RNA was purified from liver tissue or cell cultures using RNeasy Mini Kit (Qiagen, Chatsworth, CA) according to the manufacturer's instructions. Reverse transcription to cDNA was performed by using SuperScript III First-Strand Synthesis System (ThermoFisher Scientific). Quantitative real-time PCR was carried out using the QuantStudio 3 (Applied Biosystems by ThermoFisher Scientific). In a final reaction volume of 25μl, the following were added: 1× SuperMix (Platinum SYBR Green qPCR Kit; Invitrogen) cDNA and 10μM of each primer. Amplification conditions were: 50°C (2min), 95°C (5min), followed by 40 cycles of 95°C (15sec) and 60°C (30sec). The primer sequences that amplify TNF-α, IL-1β, IL-6, CXCL-2, CXCL10, MCP-1, Zc3h15, and HPRT were shown in Supplementary Table 1. The target gene expressions were calculated by their ratios to the housekeeping gene HPRT.

Western blot analysis. Protein was extracted from liver tissue or cell cultures with ice-cold protein lysis buffer (50mM Tris, 150mM NaCl, 0.1% sodium dodecyl sulfate, 1% sodium deoxycholate, 1% Triton-100). The buffer contains 1% proteinase and phosphatase inhibitor cocktails (Sigma-Aldrich, St. Louis, MO). Proteins (30 µg/sample) in SDS-loading buffer (50mM Tris, pH 7.6, 10% glycerol, 1% SDS) were subjected to 4-20% SDS-polyacrylamide gel electrophoresis (PAGE) and transferred to nitrocellulose membrane (Bio-Rad). The membrane was blocked with 5% dry milk and 0.1% Tween 20 (USB, Cleveland, OH). The nuclear and cytosolic fractions were prepared with NE-PER Nuclear and Cytoplasmic Extraction Reagents (ThermoFisher Scientific). The RIPK3, IRE1α, NOD1, RIP2, p-P65, P-65, p-JNK, JNK, Foxo1, XBP1s, Lamin B2, and β-actin (Cell Signaling Technology), Calcineurin A and Zc3h15 (ThermoFisher Scientific), and TRPM7 (Santa Cruz Biotechnology) were used. The membranes were incubated with Abs and then added Western ECL substrate mixture (Bio-Rad) for imaging with the iBright FL1000 (ThermoFisher Scientific). Relative quantities of protein were determined by comparing the β-actin expression using iBright image analysis software (ThermoFisher Scientific).

Isolation of primary hepatocytes, Kupffer cells, and bone marrow-derived macrophages. Primary hepatocytes, Kupffer cells, and BMMs from the $RIPK3^{FL/FL}$, $RIPK3^{M-KO}$, or wild-type (WT) mice were isolated as described [2]. In brief, livers were perfused in situ with warmed (37°C) HBSS solution, followed by a collagenase buffer (collagenase type IV, Sigma-Aldrich). The Perfused livers were dissected and teased through 70-µm nylon mesh cell strainers (BD Biosciences, San Jose, CA). The nonparenchymal cells (NPCs) were separated from hepatocytes by centrifuging at 50 × *g* 2min three times. The NPCs were then suspended in HBSS and layered onto a 50%/25% two-step Percoll gradient (Sigma) in a 50-ml conical centrifuge tube and centrifuged at 1800 × *g* at 4°C for 15min. The Kupffer cells in the middle layer were collected and plated to cell culture dishes in DMEM with 10% FBS, 10mM HEPES, 2mM GlutaMax, 100 U/ml penicillin, and 100 µg/ml streptomycin for 15min at 37°C. Murine bone-derived macrophages (BMMs) were generated as described [2]. In brief, bone marrow cells were removed from the femurs and tibias of the $RIPK3^{FL/FL}$, $RIPK3^{M-KO}$, or WT mice and cultured in DMEM supplemented with 10% FCS and 15% L929-conditioned medium for seven days.

Flow cytometry analysis. $1X10^5$ primary hepatocytes or liver macrophages (Kupffer cells) were washed with staining medium (phosphate-buffered saline containing 3% fetal bovine serum), and then incubated with fluorescence-conjugated antibodies. We used the Alexa Fluor 488-conjugated anti-ASGR1 polyclonal antibody (ThermoFisher Scientific) to detect hepatocytes, and Alexa Fluor 488 mouse IgG1k isotype control (BD Biosciences) was used. The BD Horizon PE-CF594 rat anti-mouse F4/80 (BD Biosciences) was used for detecting macrophages, and the PE-conjugated mouse IgG1k isotype control (BD Biosciences) was also used. The fluorescence-labeled cells were run through a flow cytometer (LSRFortessaX-20, BD Biosciences). All data were analyzed with FlowJo software (Tree Star, Inc.)

Co-culture of macrophages and primary hepatocytes. Primary hepatocytes were cultured in 6-well plates at a concentration of $4x10^5$ cells per well. After 24h, the 0.4µm-pore size

transwell inserts (Corning) containing $1x10^6$ BMMs were placed into the 6-well plate with the initially seeded hepatocytes. The co-cultures were incubated for 12h with or without adding H_2O_2 (200 μM) in the lower chamber.

ELISA assay. Cell culture supernatants were harvested for cytokine analysis. ELISA kits were used to measure the Zc3h15 (Biohippo, Gaithersburg, MD), RIPK3 (MyBioSource, San Diego, CA), TRPM7 (MyBioSource), and TNF-α (ThermoFisher Scientific) levels according to the manufacturer's instructions.

LDH activity assay. BMMs ($1x10^6$) were cultured with primary hepatocytes *(*$4x10^5$/well) for 12h with or without adding H_2O_2 (200 μM) in the lower chamber*.* The activity of lactate dehydrogenase (LDH) in the cell culture medium from the lower chamber was measured with a commercial LDH activity assay kit (Stanbio Laboratory, Boerne, TX) according to manufacturer's instructions.

Reactive oxygen species assay. ROS production in Kupffer cells was measured using the 5-(and-6)-carboxy-2',7'-difluorodihydrofluorescein diacetate (Carboxy-H2DFFDA, ThermoFisher Scientific), as described [2]. In brief, Kupffer cells ($2x10^5$) were isolated from ischemic livers and cultured on collagen-coated cover slips without or with LPS (100ng/ml) for 2h at 37°C. After washing with PBS, cells were incubated with 10μM of Carboxy-H2DFFDA. The Carboxy-H2DFFDA was converted to a green-fluorescent form when hydrolyzed by intracellular esterase and oxidized in the cells. Cells were fixed with 2% paraformaldehyde and stained with Hoechst dye. ROS produced by Kupffer cells were analyzed and quantified by fluorescence microscopy*.* Positive green fluorescent-labeled cells were counted blindly in 10 HPF/section (x200).

In vitro transfection. BMMs ($1x10^6$/well) were cultured for seven days and then and then transfected with CRISPR/Cas9-XBP1 knockout (KO), CRISPR-XBP1 activation, CRISPR/Cas9-

Zc3h15 KO, or control vector (Santa Cruz Biotechnology) by using Lipofectamine™ 3000 according to the manufacturer's instructions (ThermoFisher Scientific). After 24-48h, cells were supplemented with LPS (100 ng/ml) for an additional 6h. For lentivirus-mediated gene transfer, BMMs ($1x10^6$/well) were added with lentivirus-mediated Zc3h15 (Lv-Zc3h15) (Santa Cruz Biotechnology) or Lv-GFP control (at a multiplicity of infection 10) (Applied Biological Materials, Richmond, BC, Canada) and incubated at 37°C overnight. The medium was removed and replaced with fresh medium. After 48h, cells were harvested for *in vivo* adoptive transfer.

Immunoprecipitation analysis. BMMs after LPS stimulation were lysed in NP-40 lysis buffer (50mM Tris pH7.4, 10 mM EDTA, 150 mM NaCl, 1% NP-40, ThermoFisher Scientific) containing protease inhibitors. The lysates were incubated with XBP1s (Cell Signaling Technology), Foxo1 (Cell Signaling Technology), or control IgG and protein A/G beads at 4 ^{o}C overnight. After immunoprecipitation, the immunocomplexes were washed with lysis buffer three times and analyzed by standard immunoblot procedures.

Chromatin immunoprecipitation (ChIP). The ChIP analysis was carried out using ChIP Assay Kit (Abcam). Briefly, BMMs were treated with 1% formaldehyde for 10 min to cross-link proteins and chromatin. The reaction was stopped by adding 0.125M glycine for 5 min. Cells were washed with ice-cold PBS and then resuspended with ChIP lysis buffer for 10 min. Cell lysates were centrifuged to pellet the nuclei. The cell nuclei were resuspended in nuclei lysis buffer and then subjected to sonication for 15 min. Purified chromatin was analyzed on a 1.5 % agarose gel to analyze DNA fragment size. The sheared chromatin was immunoprecipitated with XBP1s (Cell Signaling Technology) or Foxo1 antibody (Cell Signaling Technology) overnight. As a control, the normal IgG was used as a replacement for XBP1s or Foxo1 antibody. The antibody/chromatin samples were mixed with protein A sepharose beads. Protein-DNA complexes were washed and eluted, followed by a cross-link reversal step, and the resulting DNA was purified. For sequential ChIP, sheared chromatin was first immunoprecipitated with XBP1s antibody, followed by elution

with a second immunoprecipitation using Foxo1 antibody. DNA from each immunoprecipitation reaction was examined by PCR. The primer for the Foxo1-responsive region of *Zc3h15* promoter: forward: 5'- CTGTCGCAAAGGCCACAT -3', reverse: 5'- CGATGATCTTCTCCTTCTTCTT -3'.

ChIP-sequencing (ChIP-seq). The ChIP-DNA was amplified to generate a library for sequencing. The workflow consists of fragmentation of whole-genome DNA, end repair to generate blunt ends, A-tailing, adaptor ligation, and PCR amplification. Different adaptors were used for multiplexing samples in one lane. Sequencing was performed on Illumina HiSeq3000 (Illumina, San Diego, CA) for a single read 50 run at the Technology Center for Genomics & Bioinformatics (TCGB) at UCLA. Data quality check was done on Illumina SAV. Demultiplexing was performed with the Illumina Bcl2fastq2 v 2.17 program. Reads were mapped to mouse mm10 genome using the Bowtie1, and MACS2 was used for the peak calling. ChIPseeker was used for the peak annotation. Genome browser representation files were generated by converting ChIP-seq data to bigWig format. This was done using genomeCoverageBed from bedtools v 2.17.0 to generate a bed file, then UCSC bedGraphToBigWig to convert the bed to bigWig format.

Statistical analysis. Data are expressed as mean±SD and analyzed by Permutation *t*-test and Pearson correlation. Per comparison, two-sided *p* values less than 0.05 were considered statistically significant. Multiple group comparisons were made using one-way ANOVA followed by Bonferroni's post hoc test. When groups showed unequal variances, we applied Welch's ANOVA to make various group comparisons. All analyses were used by SAS/STAT software, version 9.4.

Supplementary figures

Fig. S1

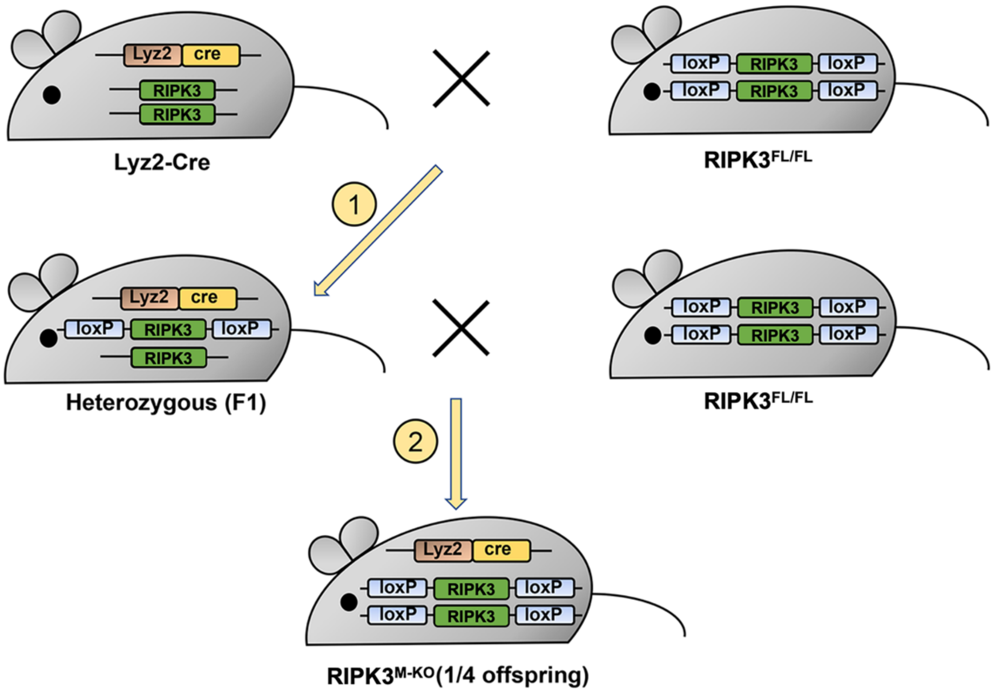

Fig. S1. Schematic illustration of generation of myeloid-specific RIPK3 knockout mice. Two steps were used to generate myeloid-specific RIPK3 KO mice. First, a homozygous loxP-flanked RIPK3 mouse is mated with a homozygous Lyz2-Cre mouse to generate the F1 mice that are heterozygous for a loxP-flanked RIPK3 allele and heterozygous for the Lyz2-cre. Next, these F1 mice were backcrossed to the homozygous loxP-flanked RIPK3 mice, resulting in the generation of myeloid-specific RIPK3 KO mice (RIPK3$^{M\text{-}KO}$, 25% of the offspring), which were homozygous for the loxP-flanked RIPK3 allele and heterozygous for the Lyz2-Cre allele.

Fig. S2

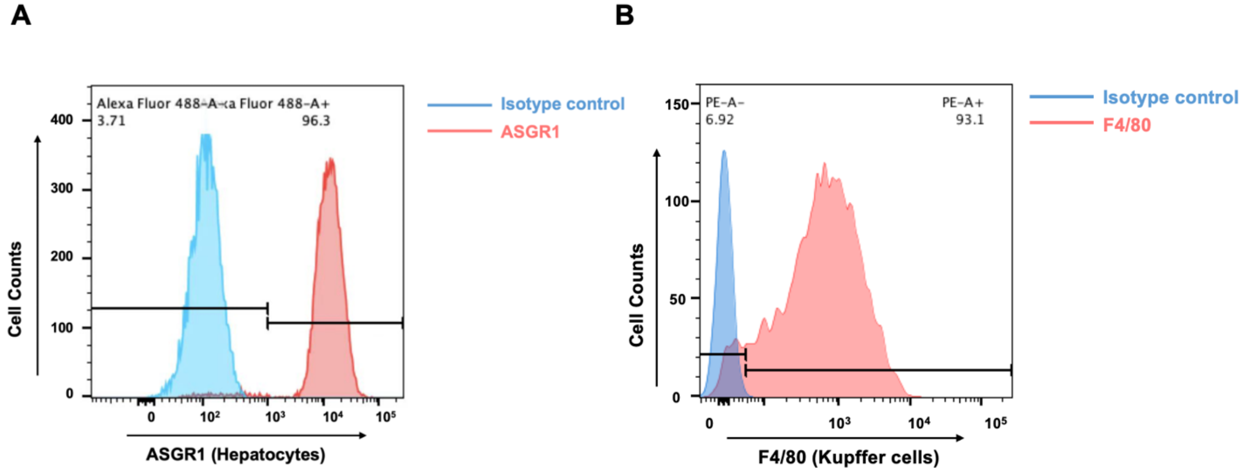

Fig. S2. Purity analysis of isolated hepatocytes and liver macrophages (Kupffer cells) by flow cytometry analysis. (A) The purity of isolated hepatocytes was analyzed by FACS with asialoglycoprotein receptors 1 (ASGR1) antibody (ThermoFisher Scientific, CL488-11739). An isotype control antibody was used (BD Biosciences, 557721). (B) The purity of isolating liver macrophages (Kupffer cells) was analyzed by FACS with F4/80 antibody (BD Biosciences, 565613). Isotype control antibody was used (BD Biosciences, 551436). *Note:* 96.3% of ASGR1+ hepatocytes (Fig. S2A) and 93.1% of F4/80+ macrophages (Fig. S2B) were shown.

Fig. S3

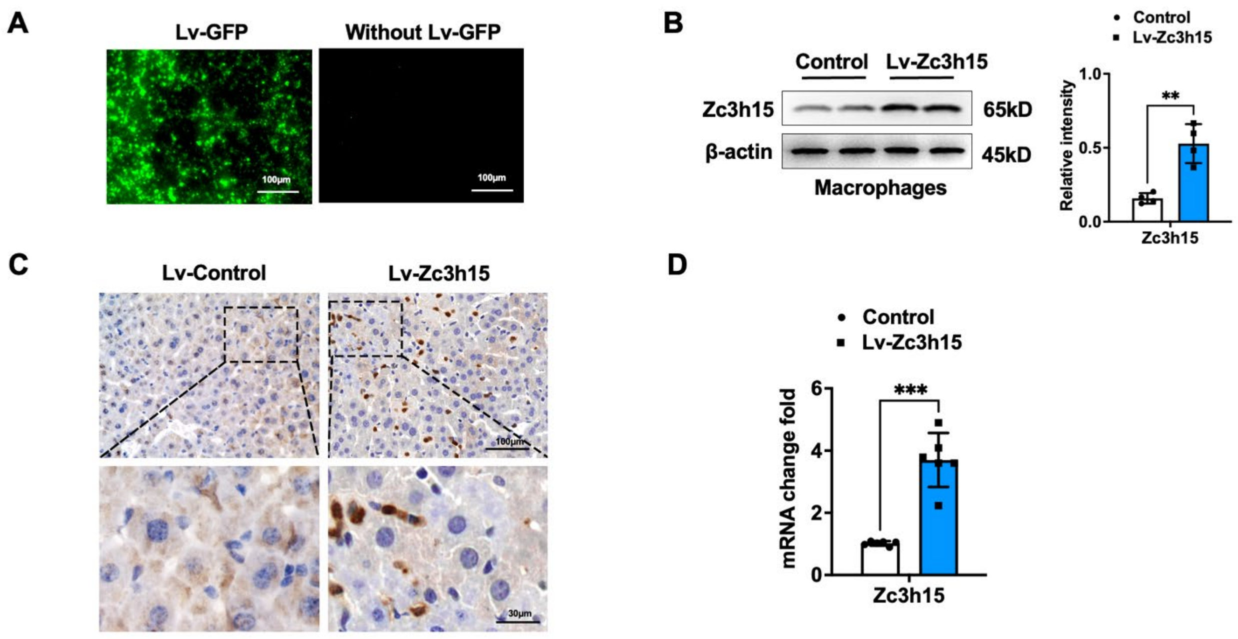

Fig. S3. **Detection of Zc3h15 expression *in vitro* and *in vivo*.** (A) To mimic the transduction efficiency of lentivirus-mediated Zc3h15 (Lv-Zc3h15) in BMMs, Lv-GFP was used to evaluate gene expression in BMMs. *Note:* more than 80% of GFP fluorescence cells were shown in Lv-GFP-transfected BMMs. (B) Western blot analysis and relative density ratio of Zc3h15 protein expression in Lv-Zc3h15-transfected BMMs. (C) Immunohistochemistry staining of Zc3h15 in IR-stressed livers 24h after tail vein injection (n=6 mice/group). Scale bars, 100μm. (D) Quantitative RT-PCR analysis of Zc3h15 mRNA levels in IR-stressed livers 24h after tail vein injection (n=6 samples/group). All Western blots represent four experiments, and the data represent the mean±SD. Statistical analysis was performed using a Permutation t-test. **p<0.01, ***p<0.001.

Fig. S4

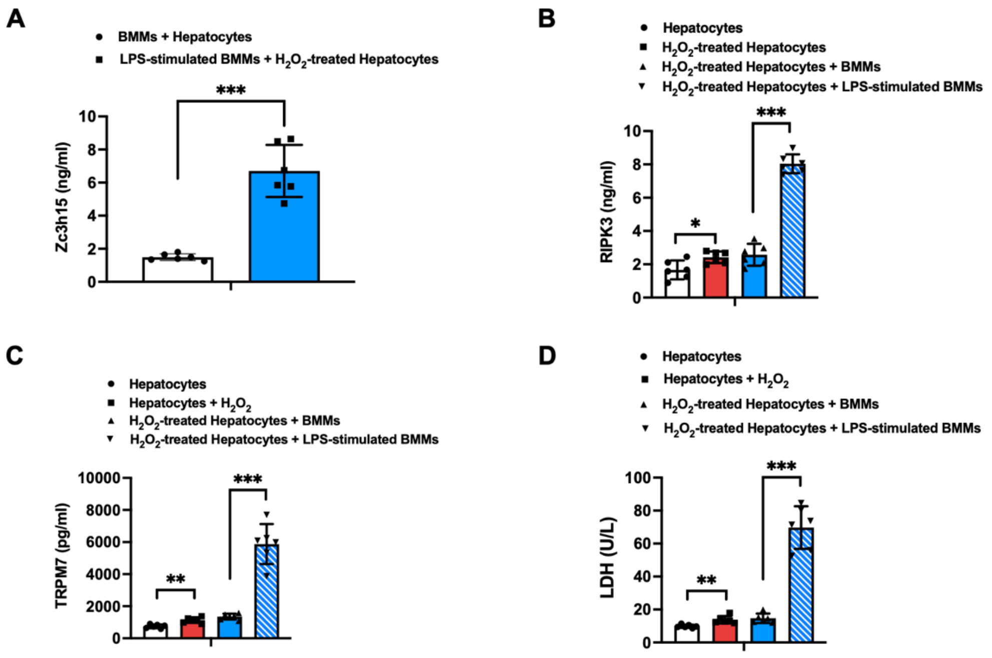

Fig. S4. ELISA analysis of Zc3h15, RIPK3, TRPM7, and LDH assay. Bone marrow-derived macrophages (BMMs) and primary hepatocytes were isolated from the RIPK3$^{FL/FL}$ mice. (A) ELISA analysis of Zc3h15 in cell supernatant after BMM/hepatocyte co-culture. (B-C) ELISA analysis of RIPK3 and TRPM7 in cell supernatant of hepatocytes, H_2O_2-treated hepatocytes alone, H_2O_2-treated hepatocytes plus co-cultured BMM with or without LPS stimulation. (D) LDH assay was performed in the cell culture medium of hepatocytes, H_2O_2-treated hepatocytes alone, H_2O_2-treated hepatocytes plus co-cultured BMM with or without LPS stimulation. The data represent the mean±SD. Statistical analysis was performed using a Permutation t-test. *$p<0.05$, **$p<0.01$, ***$p<0.001$.

Fig. S5

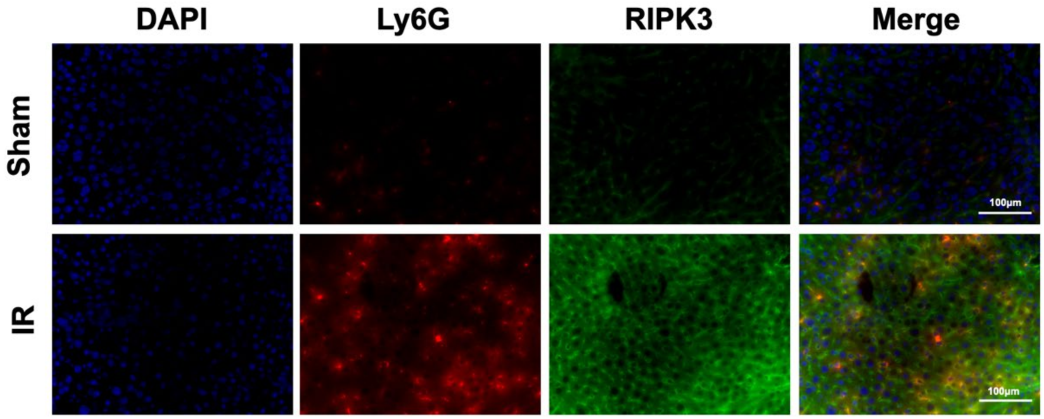

Fig. S5. RIPK3 expression in neutrophils during liver IRI. Immunofluorescence staining of RIPK3 in neutrophils from ischemic livers was performed using RIPK3 and neutrophil marker Ly6G antibodies. *Note:* RIPK3 (green) and Ly6G (red) were shown. DAPI was used to visualize nuclei (blue). Scale bars, 100μm.

Fig. S6

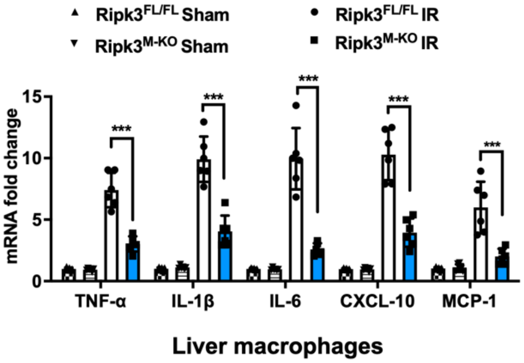

Fig. S6. Proinflammatory cytokine/chemokine expression in isolated liver macrophages. The liver macrophages were isolated from the IR-stressed RIPK3$^{FL/FL}$ and RIPK3^{M-KO} livers. The expression of cytokine/chemokine genes was analyzed by qRT-PCR. *Note:* RIPK3-deficient macrophages (RIPK3^{M-KO}) displayed reduced mRNA levels of TNF-α, IL-1β, IL-6, CXCL-10, and MCP-1 compared to the RIPK3$^{FL/FL}$ control cells. The data represent the mean±SD. Statistical analysis was performed using a Permutation t-test. ***$p<0.001$.

Fig. S7

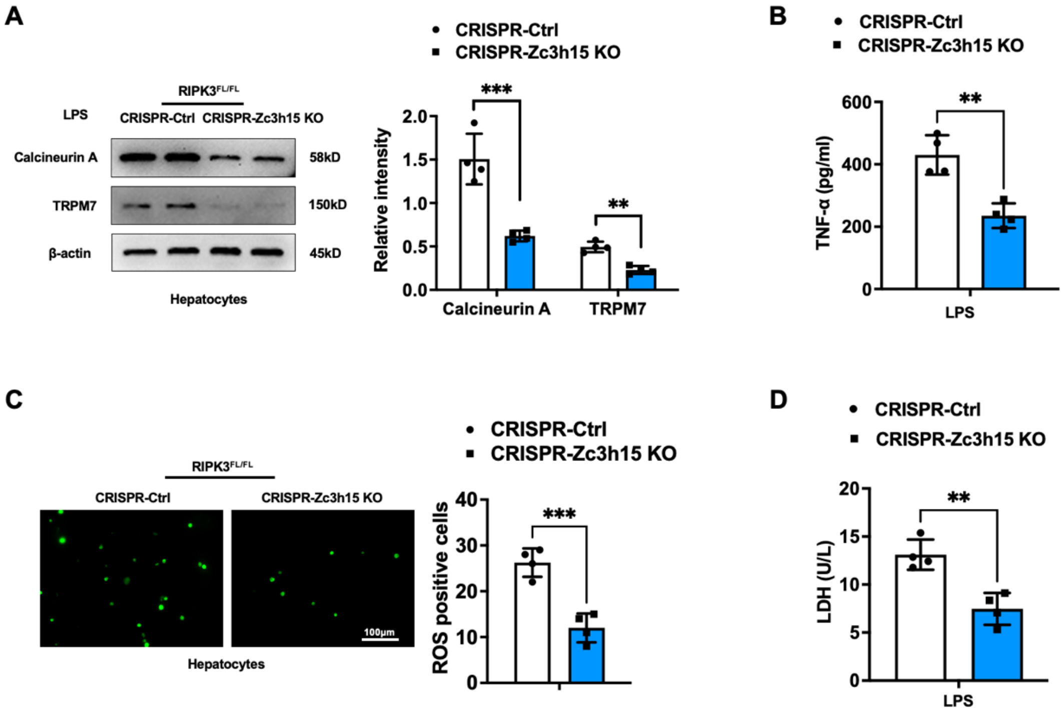

Fig. S7. LPS-stimulated Zc3h15-deficient macrophages reduce RIPK3-induced hepatocyte death after co-culture. (A) BMMs from RIPK3$^{FL/FL}$ mice were transfected with p-CRISPR-Zc3h15 KO or control vector followed by LPS (100 ng/ml) stimulation and then co-cultured with primary hepatocytes without H_2O_2 treatment. Western blot analysis and relative density ratio of Calcineurin A and TRPM7 in hepatocytes. (B) ELISA analysis of TNF-α levels in the co-culture supernatant (n=4 samples/group). (C) Detection of ROS production by Carboxy-H2DFFDA in hepatocytes. Quantification of ROS-producing hepatocytes (green) (n=4 samples/group). Scale bars, 100μm. (D) LDH release in cell medium (n=4 samples/group). All Western blots represent four experiments, and the data represent the mean±SD. Statistical analysis was performed using a Permutation t-test. **p<0.01, ***p<0.001.

Table S1: Primers used in qRT-PCR studies.

**Target genes**	**Forward primers**	**Reverse primers**
HPRT	5’-TCAACGGGGGACATAAAAGT-3’	5’-TGCATTGTTTTACCAGTGTCAA-3’
TNF-α	5’- ACGGCATGGATCTCAAAGAC-3’	5’- AGATAGCAAATCGGCTGACG-3’
IL-6	5’- GCTACCAAACTGGATATAATCAGGA -3’	5’- CCAGGTAGCTATGGTACTCCAGAA -3’
IL-1β	5’-TGTAATGAAAGACGGCACACC-3’	5’-TCTTCTTTGGGTATTGCTTGG-3’
MCP-1	5'-GAAGGAATGGGTCCAGACAT-3'	5'-ACGGGTCAACTTCACATTCA-3'
CXCL-10	5’-GCTGCCGTCATTTTCTGC-3’	5’-TCTCACTGGCCCGTCATC-3’
CXCL-2	5’-CCAACCACCAGGCTACAGG-3’	5’-GCGTCACACTCAAGCTCTG-3’
Zc3h15	5’-TTTGGTCAACAGAATCCACGTC-3’	5’-CAGCAACTACAGGTTTGAACAAC-3’

Supplementary references

[1] Li C, Sheng M, Lin Y, Xu D, Tian Y, Zhan Y, et al. Functional crosstalk between myeloid Foxo1-beta-catenin axis and Hedgehog/Gli1 signaling in oxidative stress response. Cell death and differentiation 2021;28:1705-1719.

[2] Yue S, Zhu J, Zhang M, Li C, Zhou X, Zhou M, et al. The myeloid heat shock transcription factor 1/beta-catenin axis regulates NLR family, pyrin domain-containing 3 inflammasome activation in mouse liver ischemia/reperfusion injury. Hepatology 2016;64:1683-1698.

[3] Suzuki S, Toledo-Pereyra LH, Rodriguez FJ, Cejalvo D. Neutrophil infiltration as an important factor in liver ischemia and reperfusion injury. Modulating effects of FK506 and cyclosporine. Transplantation 1993;55:1265-1272.
