# Supplementary material for: Macrophage RIPK3 triggers inflammation and cell death via the XBP1–Foxo1 axis in liver ischaemia–reperfusion injury
Source: JHEP Rep. 2023 Aug 12;5(11):100879. doi: 10.1016/j.jhepr.2023.100879 (PMC10568422; doi:10.1016/j.jhepr.2023.100879)
Supplement: Multimedia component 2 [file mmc2.docx]

**JHEP Reports**

**CTAT methods**

Tables for a “Complete, Transparent, Accurate and Timely account” (CTAT) are now mandatory for all revised submissions. The aim is to enhance the reproducibility of methods.

- Only include the parts relevant to your study
- Refer to the CTAT in the main text as ‘Supplementary CTAT Table’
- Do not add subheadings
- Add as many rows as needed to include all information
- Only include one item per row

**If the CTAT form is not relevant to your study, please outline the reasons why:**

|  |
| --- |

- 1. **Antibodies**

| **Name** | **Citation** | **Supplier** | **Cat no.** | **Clone no.** |
| --- | --- | --- | --- | --- |
| RIPK3 |  | Cell Signaling Technology | 95702 | D4G2A |
| IRE1a |  | Cell Signaling Technology | 3294 | 14C10 |
| XBP1s |  | Cell Signaling Technology | 40435 | E9V3E |
| NOD1 |  | Cell Signaling Technology | 3545 | - |
| NOD1 |  | Santa Cruz Biotechnology | sc-398696 | B-4 |
| RIP2 |  | Cell Signaling Technology | 4142 | D10B11 |
| p-P65 |  | Cell Signaling Technology | 3033S | 93H1 |
| P65 |  | Cell Signaling Technology | 8242S | D14E12 |
| p-JNK |  | Cell Signaling Technology | 4668 | 81E11 |
| JNK |  | Cell Signaling Technology | 9252 | - |
| Foxo1 |  | Cell Signaling Technology | 2880 | C29H4 |
| Foxo1 |  | Santa Cruz Biotechnology | sc-374427 | C-9 |
| Calcineurin A |  | ThermoFisher Scientific | PA5-29255 | - |
| TRPM7 |  | Santa Cruz Biotechnology | sc-271099 | H-4 |
| Zc3h15 |  | ThermoFisher Scientific | PA5-56639 | - |
| HNF4a |  | Abcam | ab201460 | EPR16885 |
| β-actin |  | Cell Signaling Technology | 5125 | 13E5 |
| LaminB2 |  | Cell Signaling Technology | 13823S | E1S1Q |
| CD11b |  | Abcam | ab8878 | M1/70 |
| Ly6G |  | Invitrogen | 14-5931-82 | RB6-8C5 |
| CD68 |  | Bio-Rad | MCA1957GA | FA-11 |
| BD Horizon™ PE-CF594 Rat Anti-Mouse F4/80 |  | BD Biosciences | 565613 | T45-2342 |
| PE-conjugated mouse IgG1κ isotype control antibody |  | BD Biosciences | 551436 | MOPC-21 |
| ASGR1 Polyclonal Antibody, CoraLite®488 |  | ThermoFisher Scientific | CL488-11739 | N/A |
| Alexa Fluor® 488 Mouse IgG1κ Isotype Control |  | BD Biosciences | 557721 | MOPC-21 |
| Alexa Fluor® 488 AffiniPure Donkey Anti-Rabbit IgG (H+L) |  | Jackson Immunoresearch | 711-545-152 | N/A |
| Alexa Fluor® 488 AffiniPure Donkey Anti-Mouse IgG (H+L) |  | Jackson Immunoresearch | 715-545-150 | N/A |
| Cy™5 AffiniPure Donkey Anti-Rat IgG (H+L) |  | Jackson Immunoresearch | 712-175-150 | N/A |
| Cy™5 AffiniPure Donkey Anti-Rabbit IgG (H+L) |  | Jackson Immunoresearch | 711-175-152 | N/A |
| Cy™5 AffiniPure Donkey Anti-Mouse IgG (H+L) |  | Jackson Immunoresearch | 715-175-150 | N/A |

- 1. **Cell lines**

| **Name** | **Citation** | **Supplier** | **Cat no.** | **Passage no.** | **Authentication test method** |
| --- | --- | --- | --- | --- | --- |
|  |  |  |  |  |  |

- 1. **Organisms**

| **Name** | **Citation** | **Supplier** | **Strain** | **Sex** | **Age** | **Overall n number** |
| --- | --- | --- | --- | --- | --- | --- |
| Wild-type mice |  | Jackson laboratory | C57BL/6J | Male | 6-8 weeks | 32 |
| RIPK3-MKO |  | Jackson laboratory | C57BL/6J | Male | 6-8 weeks | 46 |
| RIPK3-flox |  | Jackson laboratory | C57BL/6J | Male | 6-8 weeks | 40 |
| Foxo1-MKO |  | Jackson laboratory | C57BL/6J | Male | 6-8 weeks | 34 |
| Foxo1-flox |  | Jackson laboratory | C57BL/6J | Male | 6-8 weeks | 34 |

- 1. **Sequence based reagents**

| **Name** | **Sequence** | **Supplier** |
| --- | --- | --- |
| IL-6 | F: GCTACCAAACTGGATATAATCAGGA  R: CCAGGTAGCTATGGTACTCCAGAA |  |
| TNF-α | F: ACGGCATGGATCTCAAAGAC  R: AGATAGCAAATCGGCTGACG |  |
| CXCL-10 | F: GCTGCCGTCATTTTCTGC  R: TCTCACTGGCCCGTCATC |  |
| CXCL-2 | F: CCAACCACCAGGCTACAGG  R: GCGTCACACTCAAGCTCTG |  |
| IL-1β | F: TGTAATGAAAGACGGCACACC  R: TCTTCTTTGGGTATTGCTTGG |  |
| MCP-1 | F: GAAGGAATGGGTCCAGACAT  R: ACGGGTCAACTTCACATTCA |  |
| Zc3h15 | F: TTTGGTCAACAGAATCCACGTC  R: CAGCAACTACAGGTTTGAACAAC |  |
| HPRT | F: TCAACGGGGGACATAAAAGT  R: TGCATTGTTTTACCAGTGTCAA |  |

- 1. **Biological samples**

| **Description** | **Source** | **Identifier** |
| --- | --- | --- |
|  |  |  |

- 1. **Deposited data**

| **Name of repository** | **Identifier** | **Link** |
| --- | --- | --- |
|  |  |  |

- 1. **Software**

| **Software name** | **Manufacturer** | **Version** |
| --- | --- | --- |
| SAS/STAT | SAS | 9.4 |
| Image-PRO PLUS | Media Cybernetics | 6.0.0 |

- 1. **Other (*e.g*. drugs, proteins, vectors etc.)**

| CRISPR/Cas9-XBP1 KO plasmid | Santa Cruz Biotechnology | sc-423727 |
| --- | --- | --- |
| CRISPR-XBP1 Activation plasmid | Santa Cruz Biotechnology | sc-423727-ACT |
| CRISPR/Cas9-Zc3h15 KO plasmid | Santa Cruz Biotechnology | sc-427290 |
| Zc3h15 lentivirus | Santa Cruz Biotechnology | sc-427290-LAC |
| GFP lentivirus | Applied Biological Materials | Lv646 |

- 1. **Please provide the details of the corresponding methods author for the manuscript:**

| XQu2022@hotmail.com |
| --- |

**2.0 Please confirm for randomised controlled trials all versions of the clinical protocol are included in the submission. These will be published online as supplementary information.**

|  |  |  |
| --- | --- | --- |
