# Supplementary material for: Macrophage RIPK3 triggers inflammation and cell death via the XBP1–Foxo1 axis in liver ischaemia–reperfusion injury
Source: JHEP Rep. 2023 Aug 12;5(11):100879. doi: 10.1016/j.jhepr.2023.100879 (PMC10568422; doi:10.1016/j.jhepr.2023.100879)
Supplement: Multimedia component 4 [file mmc4.pdf]

Research article

JHEP|Reports

Macrophage RIPK3 triggers inflammation and cell death via the XBP1–Foxo1 axis in liver ischaemia–reperfusion injury

Authors

Xiaoye QuTao YangXiao WangDongwei XuYeping Yu, Jun Li, Longfeng Jiang, Qiang Xia, Douglas G. Farmer, Bibo Ke

Correspondence

bke@mednet.ucla.edu (B. Ke).

Graphical abstract

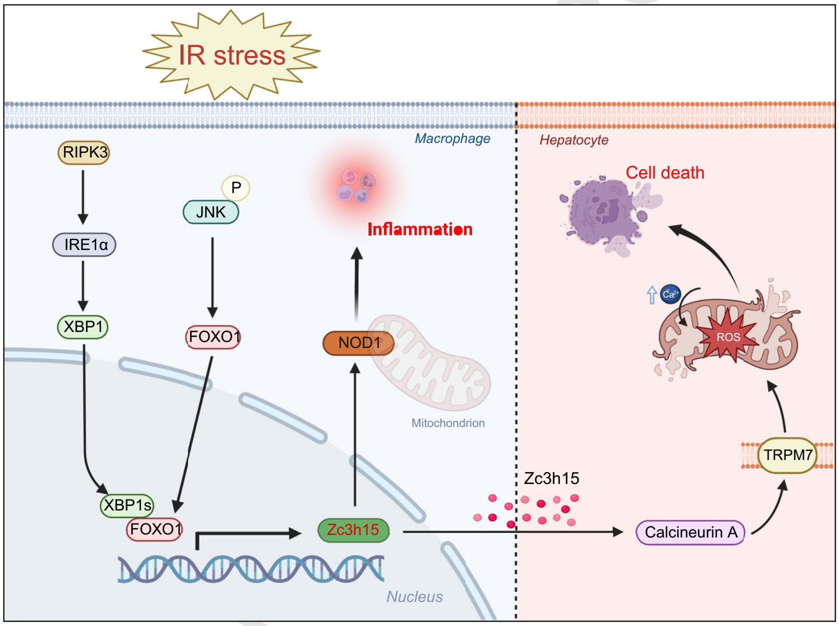

Highlights

- Macrophage RIPK3 activates the IRE1α–XBP1 pathway and Foxo1 signalling in IR-stress livers.
- Macrophage RIPK3 promotes NOD1 activation and calcineurin/TRPM7-induced hepatocyte death by triggering the XBP1–Foxo1 axis.
- The XBP1–Foxo1 interaction is essential for modulating its target gene *Zc3h15* function.
- XBP1 functions as a transcriptional coactivator of Foxo1 in regulating NOD1 and hepatocyte Calcineurin/TRPM7 activation.
- *Zc3h15* is crucial for NOD1-driven inflammation and calcineurin/TRPM7-induced cell death cascade.

Impact and implications

Macrophage RIPK3 promotes NOD1-dependent inflammation and calcineurin/TRPM7-induced cell death cascade by triggering the XBP1–Foxo1 axis and its target gene *Zc3h15*, which is crucial for activating NOD1 and calcineurin/TRPM7 function, implying the potential therapeutic targets in stress-induced liver inflammatory injury.

https://doi.org/10.1016/j.jhepr.2023.100879

UNCORRECTED PROOF

UNCORRECTED PROOF

Macrophage RIPK3 triggers inflammation and cell death via the XBP1–Foxo1 axis in liver ischaemia–reperfusion injury

Xiaoye Qu,[1,2,†] **Tao Yang**,[1,3,†] **Xiao Wang**,[1,3,†] **Dongwei Xu**,[1,2,†] Yeping Yu,[1] Jun Li,[3] Longfeng Jiang,[1,3] Qiang Xia,[2] Douglas G. Farmer,[1] Bibo Ke[1,*]

[1]The Dumont-UCLA Transplant Center, Division of Liver and Pancreas Transplantation, Department of Surgery, David Geffen School of Medicine at UCLA, Los Angeles, CA, USA; [2]Department of Liver Surgery, Renji Hospital, Shanghai Jiaotong University School of Medicine, Shanghai, China; [3]Department of Infectious Diseases, the First Affiliated Hospital, Nanjing Medical University, Nanjing, China

JHEP Reports **2023.** https://doi.org/10.1016/j.jhepr.2023.100879

Background & Aims: Receptor-interacting serine/threonine-protein kinase 3 (RIPK3) is a central player in triggering necroptotic cell death. However, whether macrophage RIPK3 may regulate NOD1-dependent inflammation and calcineurin/transient receptor potential cation channel subfamily M member 7 (TRPM7)-induced hepatocyte death in oxidative stress-induced liver inflammatory injury remains elusive.
Methods: A mouse model of hepatic ischaemia–reperfusion (IR) injury, the primary hepatocytes, and bone marrow-derived macrophages were used in the myeloid-specific RIPK3 knockout (RIPK3^{M-KO}) and RIPK3-proficient (RIPK3$^{FL/FL}$) mice.
Results: RIPK3^{M-KO} diminished IR stress-induced liver damage with reduced serum alanine aminotransferase/aspartate aminotransferase levels, macrophage/neutrophil infiltration, and pro-inflammatory mediators compared with the RIPK3$^{FL/FL}$ controls. IR stress activated RIPK3, inositol-requiring transmembrane kinase/endoribonuclease 1α (IRE1α), x-box binding protein 1 (XBP1), nucleotide-binding oligomerisation domain-containing protein 1 (NOD1), NF-κB, forkhead box O1 (Foxo1), calcineurin A, and TRPM7 in ischaemic livers. Conversely, RIPK3^{M-KO} depressed IRE1α, XBP1, NOD1, calcineurin A, and TRPM7 activation with reduced serum tumour necrosis factor α (TNF-α) levels. Moreover, Foxo1^{M-KO} alleviated IR-induced liver injury with reduced NOD1 and TRPM7 expression. Interestingly, chromatin immunoprecipitation coupled with massively parallel sequencing revealed that macrophage Foxo1 colocalised with XBP1 and activated its target gene *Zc3h15* (zinc finger CCCH domain-containing protein 15). Activating macrophage XBP1 enhanced *Zc3h15*, NOD1, and NF-κB activity. However, disruption of macrophage *Zc3h15* inhibited NOD1 and hepatocyte calcineurin/TRPM7 activation, with reduced reactive oxygen species production and lactate dehydrogenase release after macrophage/hepatocyte coculture. Furthermore, adoptive transfer of *Zc3h15*-expressing macrophages in RIPK3^{M-KO} mice augmented IR-triggered liver inflammation and cell death.
Conclusions: Macrophage RIPK3 activates the IRE1α–XBP1 pathway and Foxo1 signalling in IR-stress livers. The XBP1–Foxo1 interaction is essential for modulating target gene *Zc3h15* function, which is crucial for the control of NOD1 and calcineurin-mediated TRPM7 activation. XBP1 functions as a transcriptional coactivator of Foxo1 in regulating NOD1-driven liver inflammation and calcineurin/TRPM7-induced cell death. Our findings underscore a novel role of macrophage RIPK3 in stress-induced liver inflammation and cell death, implying the potential therapeutic targets in liver inflammatory diseases.
Impact and implications: Macrophage RIPK3 promotes NOD1-dependent inflammation and calcineurin/TRPM7-induced cell death cascade by triggering the XBP1–Foxo1 axis and its target gene *Zc3h15*, which is crucial for activating NOD1 and calcineurin/TRPM7 function, implying the potential therapeutic targets in stress-induced liver inflammatory injury.
© 2023 The Author(s). Published by Elsevier B.V. on behalf of European Association for the Study of the Liver (EASL). This is an open access article under the CC BY license (http://creativecommons.org/licenses/by/4.0/).

Introduction

Hepatic inflammation and injury initiated by ischaemia and reperfusion (IR) are the leading cause of hepatic dysfunction and failure following liver transplantation, resection, and haemorrhagic shock.[1] Endoplasmic reticulum (ER) and oxidative stress are the most important pathological mechanisms in IR injury (IRI), which causes inflammation and cell death through multiple pathways.[2] IR stress activates macrophages and releases reactive oxygen species (ROS), which primes the innate immune system and initiates liver inflammatory injury.[1,3–5]

Nucleotide-binding oligomerisation domain-containing protein 1 (NOD1), a member of the NOD-like receptor family of cytosolic pattern recognition receptors, has been recognised as a crucial sensor of the innate immune system in response to invading pathogens and stress signals.[6,7] Upon activation, NOD1 mediates distinct cellular responses. It initiates signal transduction mechanisms, including stimulation of NF-κB, mitogen-

Keywords: ER stress; Innate immunity; IRE1α; XBP1; Foxo1; Reactive oxygen species; Necroptosis; Liver inflammation.
Received 21 November 2022; received in revised form 12 May 2023; accepted 22 July 2023; available online 12 August 2023
[†] These authors contributed equally to this work.
* Corresponding author. Address: The Dumont-UCLA Transplant Center, Division of Liver and Pancreas Transplantation, Department of Surgery, David Geffen School of Medicine at UCLA, 77-120 CHS, 10833 Le Conte Ave, Los Angeles, CA 90095, USA. Tel.: +1-310-825-7444; Fax: +1-310-267-2367.
E-mail address: bke@mednet.ucla.edu (B. Ke).

activated protein kinases (MAPKs), interferon regulatory factors, and programmed cell death. NOD1 stimulation recruited inflammatory cells and induced chemokine production.[8] Activation of NOD1 increased macrophage accumulation and contributed to the progression of cardiovascular inflammation,[9] whereas disruption of NOD1 ameliorated vascular inflammation-mediated injury.[10] Moreover, cellular stress promoted NOD1-dependent inflammation by triggering NF-κB activation.[11] ROS may induce NOD1 activation with pro-inflammatory gene expression in response to ER or oxidative stress.[6,12] These results indicate that NOD1 signalling is critical in mediating innate immune activation during a stress-induced inflammatory response.

Receptor-interacting serine/threonine-protein kinase 3 (RIPK3) contains a C-terminal domain unique from other RIP family members. The encoded protein is primarily localised to the cytoplasm.[13] RIPK3 can form a complex with tumour necrosis factor receptor 1 (TNFR1) to induce necroptosis by interaction with RIPK1 and mixed lineage kinase domain-like pseudokinase (MLKL).[14,15] Indeed, the initiation of necroptosis is involved in the ligation of TNFR1, which binds to tumour necrosis factor (TNF).[15] Activation of RIPK3 phosphorylates the pseudokinase MLKL, which is translocated into the inner leaflet of the plasma membrane.[16] Moreover, a transient disruption in membrane integrity results in an abrupt calcium influx. Increased mitochondrial Ca^{2+} accumulation could result in cell death mediated by transient receptor potential cation channel subfamily M member 7 (TRPM7) under cell stress conditions.[17,18] Interestingly, RIPK3 can also trigger inflammatory signalling pathways, especially in tissue injury and sterile inflammation.[19] Activation of RIPK3 promoted inflammatory injury in non-alcoholic steatohepatitis,[20] whereas disruption of RIPK3 dampened inflammation and non-alcoholic steatohepatitis progression.[21] Moreover, RIPK3 interacted with Toll-like receptor 4 (TLR4) to activate NF-κB and pro-inflammatory cytokine production in response to cell stress.[22] Conversely, RIPK3 deletion prevented ER stress-induced cell death.[23] Although the role of RIPK3-mediated necroptosis and inflammation has been characterised, we know very little about the mechanism of macrophage RIPK3 in regulating NOD1 function and TRPM7-induced cell death in IR stress-induced liver inflammatory injury.

Here, we identify a novel regulatory mechanism of macrophage RIPK3 on NOD1 function and the TRPM7-mediated cell death pathway in IR stress-induced liver inflammation. We demonstrate that macrophage RIPK3 modulates NOD1 and calcineurin-mediated TRPM7 activation by controlling the x-box binding protein 1 (XBP1)–forkhead box O1 (Foxo1) axis and its target gene *Zc3h15* (zinc finger CCCH domain-containing protein 15), which is critical in triggering NOD1-driven inflammatory responses and calcineurin/TRPM7-induced hepatocyte death in IR-stressed livers.

Materials and methods

Animals

The floxed RIPK3 ($RIPK3^{FL/FL}$) mice (B6;129-*RIPK3^tm1.1Fkmc^*/J) and the mice expressing Cre recombinase under the control of the lysozyme 2 (Lyz2) promoter (LysM-Cre) were obtained from The Jackson Laboratory (Bar Harbor, ME, USA). The targeting vector is designed to insert a loxP site and a Flippase recognition target (FRT)-flanked neomycin resistance (neo) upstream of exon 10. An enhanced green fluorescent protein sequence, followed by a second loxP site, is inserted at the end of the coding region. Flp-mediated recombination removed the FRT-flanked neo cassette. This strain was maintained on a mixed 129 and C57BL/6 genetic background. To generate myeloid-specific RIPK3 knockout ($RIPK3^{M-KO}$) mice, a homozygous loxP-flanked RIPK3 mouse was mated with a homozygous Lyz2-Cre mouse to create the F1 mice that were heterozygous for a loxP-flanked RIPK3 allele and heterozygous for the Lyz2-Cre. The F1 mice were then backcrossed to the homozygous loxP-flanked RIPK3 mice, resulting in the generation of $RIPK3^{M-KO}$ mice (25% of the offspring), which were homozygous for the loxP-flanked RIPK3 allele and heterozygous for the Lyz2-Cre allele (Fig. S1). The myeloid-specific Foxo1 knockout ($Foxo1^{M-KO}$) mice were generated as described.[1] Mouse genotyping was performed using a standard protocol with primers described in the JAX Genotyping Protocol Database. Male mice at 6–8 weeks of age were used in all experiments. This study was performed in strict accordance with the recommendations in the *Guide for the Care and Use of Laboratory Animals* published by the National Institutes of Health. Animal protocols were approved by the Institutional Animal Care and Use Committee of The University of California at Los Angeles.

Mouse liver IRI model

We used an established mouse model of warm hepatic ischaemia (90 min) followed by reperfusion (6 h).[3] Mice were injected with heparin (100 U/kg), and an atraumatic clip was used to interrupt the arterial/portal venous blood supply to the cephalad liver lobes. After 90 min of ischaemia, the clip was removed, and mice were sacrificed at 6 h of reperfusion. Some animals were injected via tail vein with Zc3h15-expressing bone marrow-derived macrophages (BMMs) or control cells (1×10^6 cells in 0.1 ml of PBS/mouse) 24 h before ischaemia.

Statistical analysis

Data are expressed as mean ± SD and analysed using a permutation *t* test and Pearson correlation. Per comparison, two-sided *p* values less than 0.05 were considered statistically significant. Multiple group comparisons were made using one-way ANOVA followed by Bonferroni's *post hoc* test. When groups showed unequal variances, we applied Welch's ANOVA to make various group comparisons. All analyses were performed using SAS/STAT software, version 9.4.

For further details regarding the materials and methods used, please refer to the Supplementary CTAT Table and Supplementary information.

Results

Myeloid-specific RIPK3 deficiency alleviates IR-induced liver damage and diminishes macrophage/neutrophil infiltration and pro-inflammatory mediators in IR-stressed liver

The myeloid-specific RIPK3-deficient ($RIPK3^{M-KO}$) and RIPK3-proficient ($RIPK3^{FL/FL}$) mice were subjected to 90 min of warm ischaemia followed by 6 h of reperfusion. The primary hepatocytes and liver macrophages (Kupffer cells) were isolated from these ischaemic livers. Indeed, the RIPK3 expression was undetectable in liver macrophages but not in hepatocytes of $RIPK3^{M-KO}$ mice (Fig. 1A). The liver damage was assessed using Suzuki's histological grading of liver IRI[24] (Fig. 1B). The $RIPK3^{FL/FL}$ livers showed severe oedema, sinusoidal congestion, and extensive hepatocellular necrosis. In contrast, The $RIPK3^{M-KO}$ livers displayed mild to moderate oedema, sinusoidal congestion, and mild necrosis (Fig. 1B). The liver function was measured by the serum alanine aminotransferase (sALT) and serum aspartate aminotransferase (sAST) levels. $RIPK3^{M-KO}$ significantly decreased sALT and sAST levels at 6 h post liver

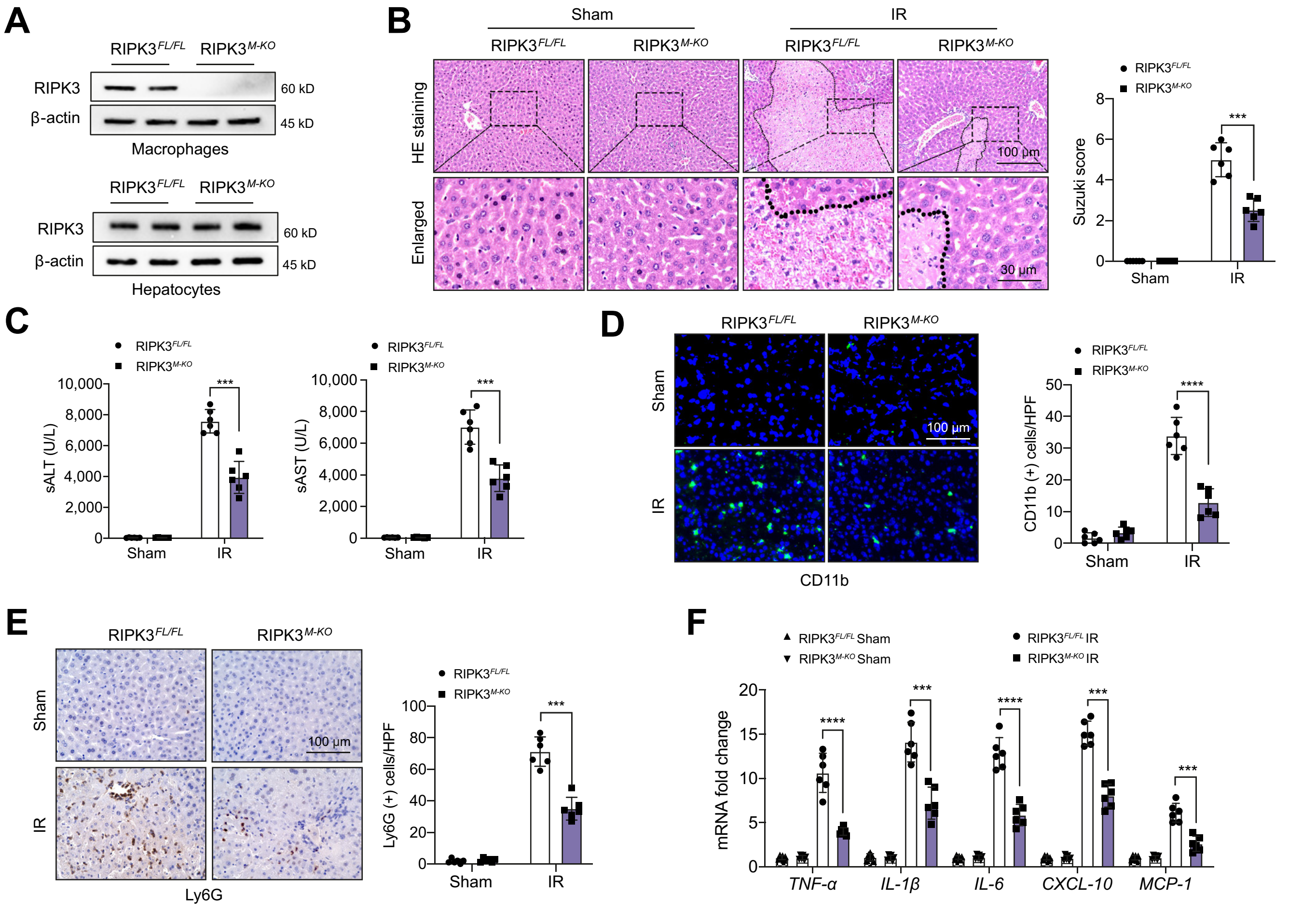

Fig. 1. Myeloid-specific RIPK3 deficiency alleviates IR-induced liver damage and diminishes macrophage/neutrophil infiltration and pro-inflammatory mediators in IR-stressed liver. The RIPK3$^{FL/FL}$ and RIPK3$^{M\text{-}KO}$ mice were subjected to 90 min of partial liver warm ischaemia, followed by 6 h of reperfusion. (A) The RIPK3 expression was detected in hepatocytes and liver macrophages from IR-stressed livers by Western blot assay. Representative of four experiments. (B) Representative histological staining (H&E) of ischaemic liver tissue (n = 6 mice/group) and Suzuki's histological score. Scale bars, 200 and 30 μm. (C) Liver function was evaluated by sALT and sAST levels (IU/L) (n = 6 samples/group). (D) Immunofluorescence staining of CD11b$^+$ macrophages in ischaemic livers (n = 6 mice/group). Quantification of CD11b$^+$ macrophages. Scale bars, 100 μm. (E) Immunohistochemistry staining of Ly6G$^+$ neutrophils in ischaemic livers (n = 6 mice/group). Quantification of Ly6G$^+$ neutrophils. Scale bars, 100 μm. (F) qRT-PCR analysis of TNF-α, IL-6, CXCL-10, and MCP-1 mRNA levels in ischaemic livers (n = 6 samples/group). All data represent the mean ± SD. Statistical analysis was performed using a permutation *t* test. ***p <0.005, ****p <0.001. CXCL-10, C–X–C motif chemokine ligand 10; HPF, high-power field; IR, ischaemia and reperfusion; MCP-1, monocyte chemoattractant protein 1; qRT-PCR, quantitative reverse transcription PCR; RIPK3, receptor-interacting serine/threonine-protein kinase 3; sALT, serum alanine aminotransferase; sAST, serum aspartate aminotransferase; TNF-α, tumour necrosis factor α.

reperfusion compared with the RIPK3$^{FL/FL}$ controls (Fig. 1C). Moreover, RIPK3$^{M\text{-}KO}$ markedly decreased accumulation of CD11b$^+$ macrophages (Fig. 1D) and Ly6G$^+$ neutrophils (Fig. 1E), with reduced mRNA levels of tumour necrosis factor α (TNF-α), IL-1β, IL-6, C–X–C motif chemokine ligand 10 (CXCL-10), and monocyte chemoattractant protein 1 (MCP-1) in ischaemic livers and liver macrophages (Fig. 1F and Fig. S6). These results suggest that RIPK3 contributes to IR stress-induced liver inflammation and injury.

Myeloid-specific RIPK3 deficiency regulates the IRE1α–XBP1 pathway and inhibits NOD1 and calcineurin/TRPM7 activation in IR-stressed liver

As inositol-requiring transmembrane kinase/endoribonuclease 1α (IRE1α) and nuclear transcription factor XBP1 play a critical role in tissue inflammation in response to ER stress,[25] we next analysed whether RIPK3 may affect the IRE1α–XBP1 pathway in IR-stressed livers. Indeed, IR stress activated RIPK3 and augmented IRE1α and spliced XBP1 (XBP1s) expression in ischaemic livers (Fig. 2A). Immunofluorescence staining showed that IR stress increased RIPK3 expression in liver macrophages from the ischaemic livers (Fig. 2B). As expected, IR stress induced NOD1 and P65 NF-κB activation (Fig. 2A). Strikingly, IR stress activated c-Jun N-terminal kinase (JNK) and increased nuclear Foxo1 expression in ischaemic livers (Fig. 2C). Moreover, increased nuclear XBP1s and Foxo1 expression were observed in Kupffer cells from ischaemic livers (Fig. 2D). Unlike RIPK3$^{FL/FL}$ controls, RIPK3$^{M\text{-}KO}$ reduced IRE1α, XBP1s, NOD1, and p-P65 protein expression in IR-stressed livers (Fig. 2E), with reduced serum levels of TNF-α (Fig. 2F). More importantly, RIPK3$^{M\text{-}KO}$ inhibited calcineurin A and TRPM7 activation in IR-stressed livers (Fig. 2E). This result was further confirmed by immunofluorescence staining, which showed that RIPK3$^{M\text{-}KO}$ decreased TRPM7 expression in IR-stressed livers (Fig. 2G). These

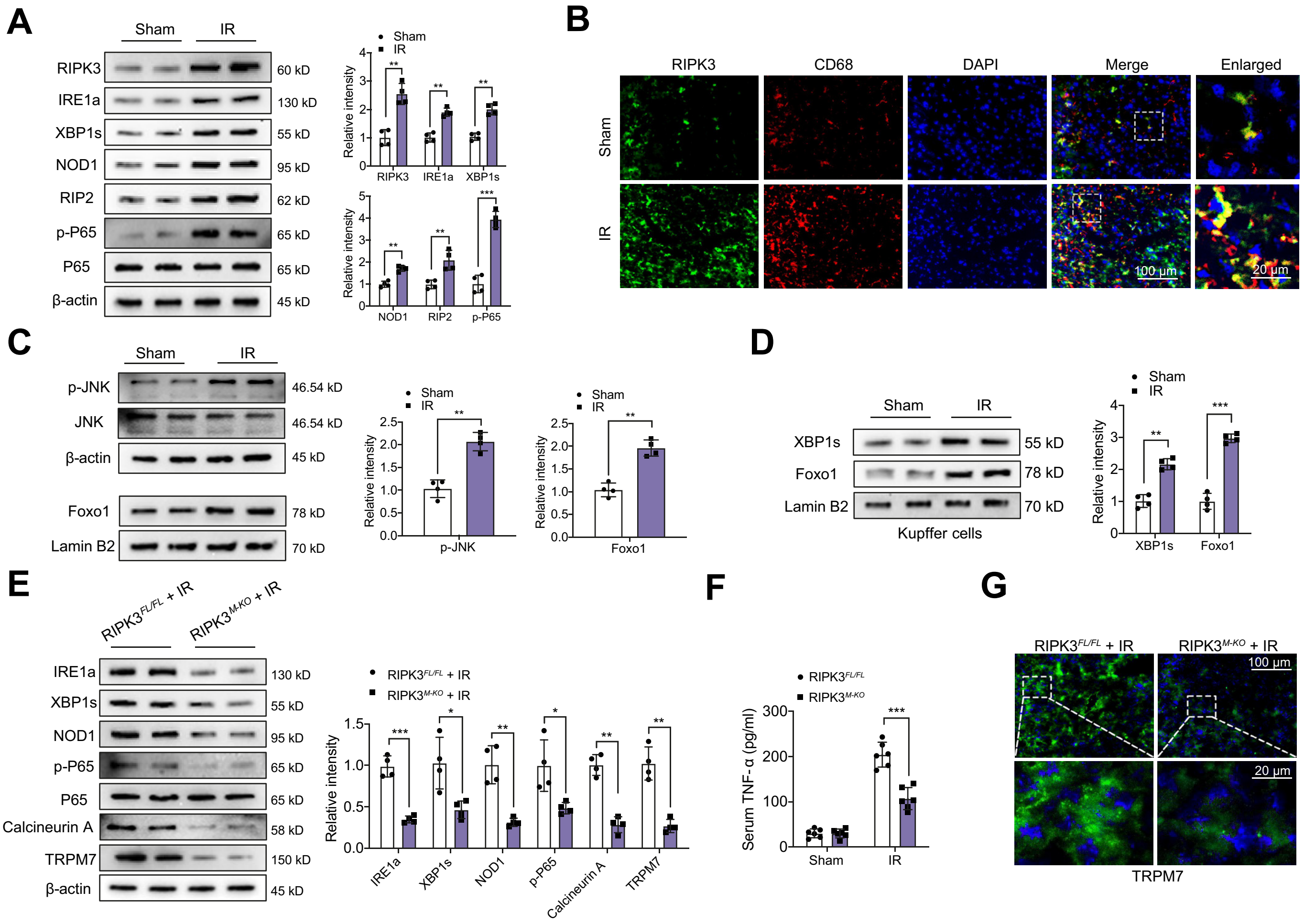

Fig. 2. Myeloid-specific RIPK3 deficiency regulates the IRE1α–XBP1 pathway and inhibits NOD1 and calcineurin/TRPM7 activation in IR-stressed liver. The WT, RIPK3$^{FL/FL}$, and RIPK3^{M-KO} mice were subjected to 90 min of partial liver warm ischaemia, followed by 6 h of reperfusion. (A) Western-assisted analysis and relative density ratio of RIPK3, IRE1α, XBP1s, NOD1, receptor (TNFRSF)-interacting serine-threonine kinase 2 (RIP2), p-P65, and P-65 in the WT livers after IR stress. (B) Immunofluorescence staining of RIPK3 and CD68 in ischaemic livers (n = 6 mice/group). Scale bars, 100 and 20 μm. (C) Western-assisted analysis and relative density ratio of p-JNK, JNK, and nuclear Foxo1 in the WT livers after IR stress. (D) The Kupffer cells were isolated from the WT livers after IR stress. Western-assisted analysis and relative density ratio of nuclear XBP1s and Foxo1 in Kupffer cells. (E) Western-assisted analysis and relative density ratio of IRE1α, XBP1s, NOD1, p-P65, P-65, calcineurin A, and TRPM7 in the RIPK3$^{FL/FL}$ and RIPK3^{M-KO} livers after IR stress. (F) ELISA analysis of serum TNF-α levels in the RIPK3$^{FL/FL}$ and RIPK3^{M-KO} mice after IR stress (n = 6 samples/group). (G) Immunofluorescence staining of TRPM7 in the RIPK3$^{FL/FL}$ and RIPK3^{M-KO} livers after IR stress (n = 6 mice/group). Scale bars, 100 and 20 μm. All Western blots represent four experiments, and the data represent the mean ± SD. Statistical analysis was performed using a permutation *t* test. $^{*}p$ <0.05, $^{**}p$ <0.01, $^{***}p$ <0.005. Foxo1, forkhead box O1; IR, ischaemia and reperfusion; IRE1α, inositol-requiring transmembrane kinase/endoribonuclease 1α; JNK, c-Jun N-terminal kinase; NOD1, nucleotide-binding oligomerisation domain-containing protein 1; RIPK3, receptor-interacting serine/threonine-protein kinase 3; TNF-α, tumour necrosis factor α; TRPM7, transient receptor potential cation channel subfamily M member 7; WT, wild-type; XBP1, x-box binding protein 1; *XBP1s*, spliced XBP1.

data suggest that macrophage RIPK3 is key in activating the IRE1α–XBP1 pathway and promoting NOD1 and calcineurin/TRPM7 functions in IR stress-induced liver injury.

Disruption of myeloid Foxo1 ameliorates liver injury and dampens NOD1 and calcineurin/TRPM7 activation in IR-stressed livers

As IR stress activated macrophage Foxo1 signalling, we then determined the role of Foxo1 in IR-stressed livers. Unlike in Foxo1$^{FL/FL}$ controls, Foxo1^{M-KO} alleviated IR-induced liver damage (Fig. 3A), with reduced sALT/sAST levels (Fig. 3B) and CD11b^{+} macrophage (Fig. 3C) and Ly6G^{+} neutrophil (Fig. 3D) accumulation. Moreover, Foxo1^{M-KO} inhibited NOD1, p65, calcineurin A, and TRPM7 activation (Fig. 3E), with reduced expression of TNF-α, IL-1β, IL-6, CXCL-10, and MCP-1 (Fig. 3F) in IR-stressed livers, compared with Foxo1$^{FL/FL}$ controls. These results imply that Foxo1 signalling is involved in NOD1-driven liver inflammation and calcineurin/TRPM7-induced cell death during liver IRI.

XBP1 interacts with Foxo1 and regulates NOD1 activation in macrophages

As IR stress activated the IRE1α–XBP1 pathway and Foxo1 signalling in ischaemic livers, we examined whether there is a crosstalk between the IRE1α–XBP1 pathway and Foxo1 signalling during an inflammatory response. Indeed, immunofluorescence staining showed increased nuclear XBP1s (Fig. 4A) and Foxo1 (Fig. 4B) expression in lipopolysaccharide (LPS)-stimulated BMMs. Interestingly, XBP1s and Foxo1 were colocalised in the nucleus (Fig. 4C). This result was further confirmed by Western blot assay, which showed that increased nuclear XBP1s and Foxo1 protein

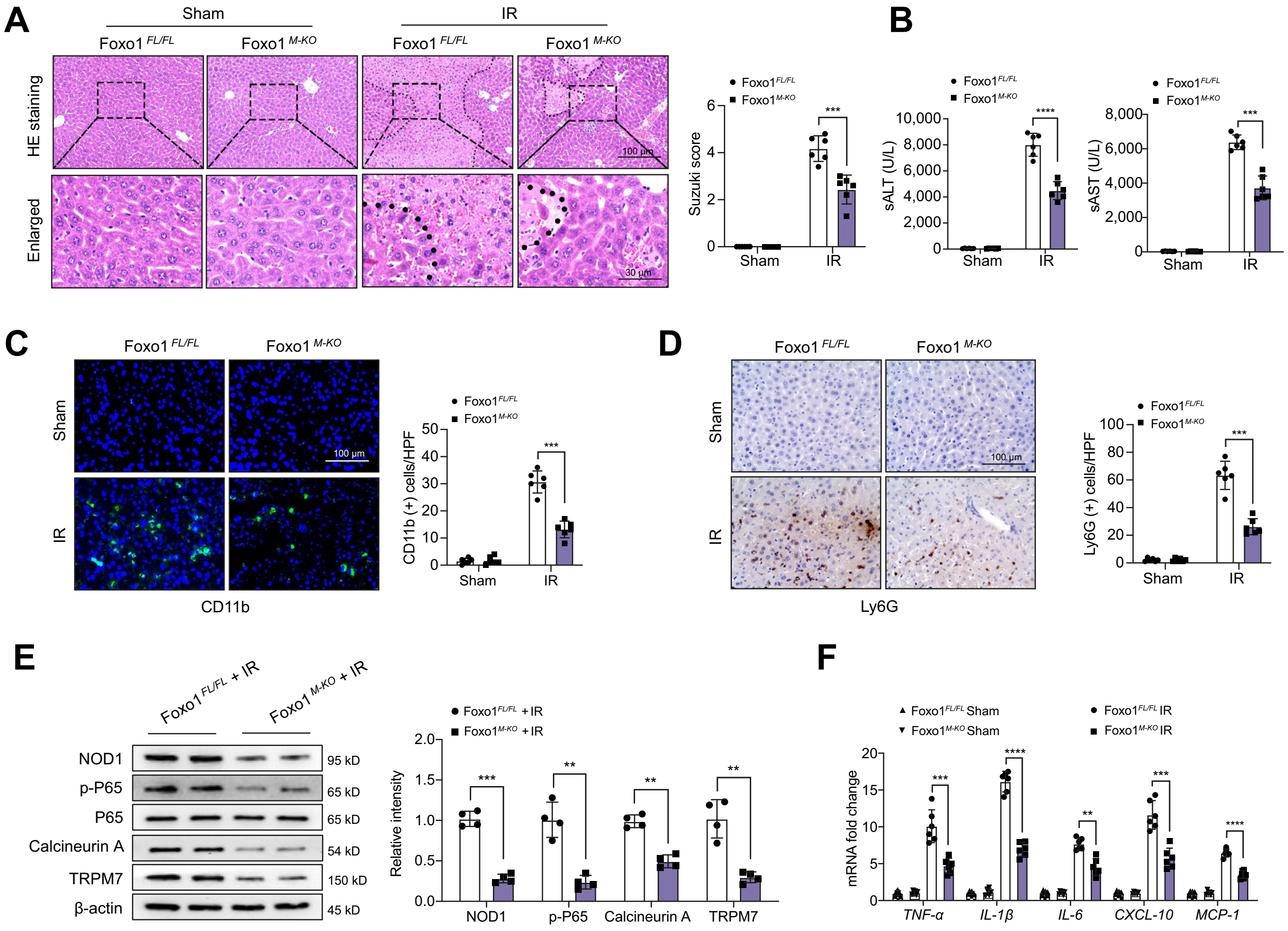

Fig. 3. Disruption of myeloid Foxo1 ameliorates liver injury and dampens NOD1 and calcineurin/TRPM7 activation in IR-stressed livers. The Foxo1$^{FL/FL}$ and Foxo1^{M-KO} mice were subjected to 90 min of partial liver warm ischaemia, followed by 6 h of reperfusion. (A) Representative histological staining (H&E) of ischaemic liver tissue (n = 6 mice/group) and Suzuki's histological score. Scale bars, 100 and 30 μm. (B) Liver function was evaluated by sALT and sAST levels (IU/L) (n = 6 samples/group). (C) Immunofluorescence staining of CD11b$^+$ macrophages in ischaemic livers (n = 6 mice/group). Quantification of CD11b$^+$ macrophages, Scale bars, 100 μm. (D) Immunohistochemistry staining of Ly6G$^+$ neutrophils in ischaemic livers (n = 6 mice/group). Quantification of Ly6G$^+$ neutrophils. Scale bars, 100 μm. (E) Western-assisted analysis and relative density ratio of NOD1, p-P65, P-65, calcineurin A, and TRPM7 in the Foxo1$^{FL/FL}$ and Foxo1^{M-KO} livers after IR stress. (F) qRT-PCR analysis of TNF-α, IL-6, CXCL-10, and MCP-1 mRNA levels in ischaemic livers (n = 6 samples/group). All immunoblots represent four experiments, and data represent the mean ± SD. Statistical analysis was performed using a Permutation t-test. $^{**}p$ <0.01, $^{***}p$ <0.005, $^{****}p$ <0.001. CXCL-10, C–X–C motif chemokine ligand 10; Foxo1, forkhead box O1; IR, ischaemia and reperfusion; MCP-1, monocyte chemoattractant protein 1; NOD1, nucleotide-binding oligomerisation domain-containing protein 1; qRT-PCR, quantitative reverse transcription PCR; sALT, serum alanine aminotransferase; sAST, serum aspartate aminotransferase; TNF-α, tumour necrosis factor α; TRPM7, transient receptor potential cation channel subfamily M member 7.

expression in macrophages after LPS stimulation (Fig. 4D). We next used a co-immunoprecipitation assay to detect the interaction between XBP1s and Foxo1 under inflammatory conditions. Strikingly, co-immunoprecipitation analysis revealed that XBP1s bound to endogenous Foxo1 in macrophages after LPS stimulation (Fig. 4E). Moreover, disruption of RIPK3 depressed NOD1 and P65 activation in LPS-stimulated RIPK3^{M-KO} macrophages (Fig. 4F). Hence, these results suggest that the macrophage XBP1s–Foxo1 axis is crucial for the NOD1-driven inflammatory response in RIPK3-mediated immune regulation.

The XBP1–Foxo1 axis targets *Zc3h15* and modulates NOD1-driven inflammatory response in macrophages

To explore the potential mechanism of the XBP1–Foxo1 axis in the modulation of NOD1 function in macrophages, we performed Foxo1 chromatin immunoprecipitation (ChIP) coupled to massively parallel sequencing (ChIP-seq). Indeed, Foxo1 ChIP-seq peaks were identified within the *Zc3h15* gene. One was located in the promoter region, and the others were within the intron or exon (Fig. 5A). To validate the ChIP-seq peak in the *Zc3h15* promoter region, ChIP-PCR was performed using Foxo1 and XBP1s antibodies in macrophages. The primer was designed to detect the Foxo1 DNA-binding site in the *Zc3h15* promoter by PCR analysis. The sequential ChIPs showed that XBP1s and Foxo1 were bound to the Foxo1-binding motif in the Foxo1–chromatin complex (Fig. 5B), confirming that XBP1s and Foxo1 are present at the same promoter region of *Zc3h15*. Hence, Zc3h15 is a target gene regulated by the XBP1s–Foxo1 complex. Moreover, RIPK3^{M-KO} diminished Zc3h15 mRNA levels (Fig. 5C) and protein expression (Fig. 5D) in LPS-stimulated macrophages compared with the

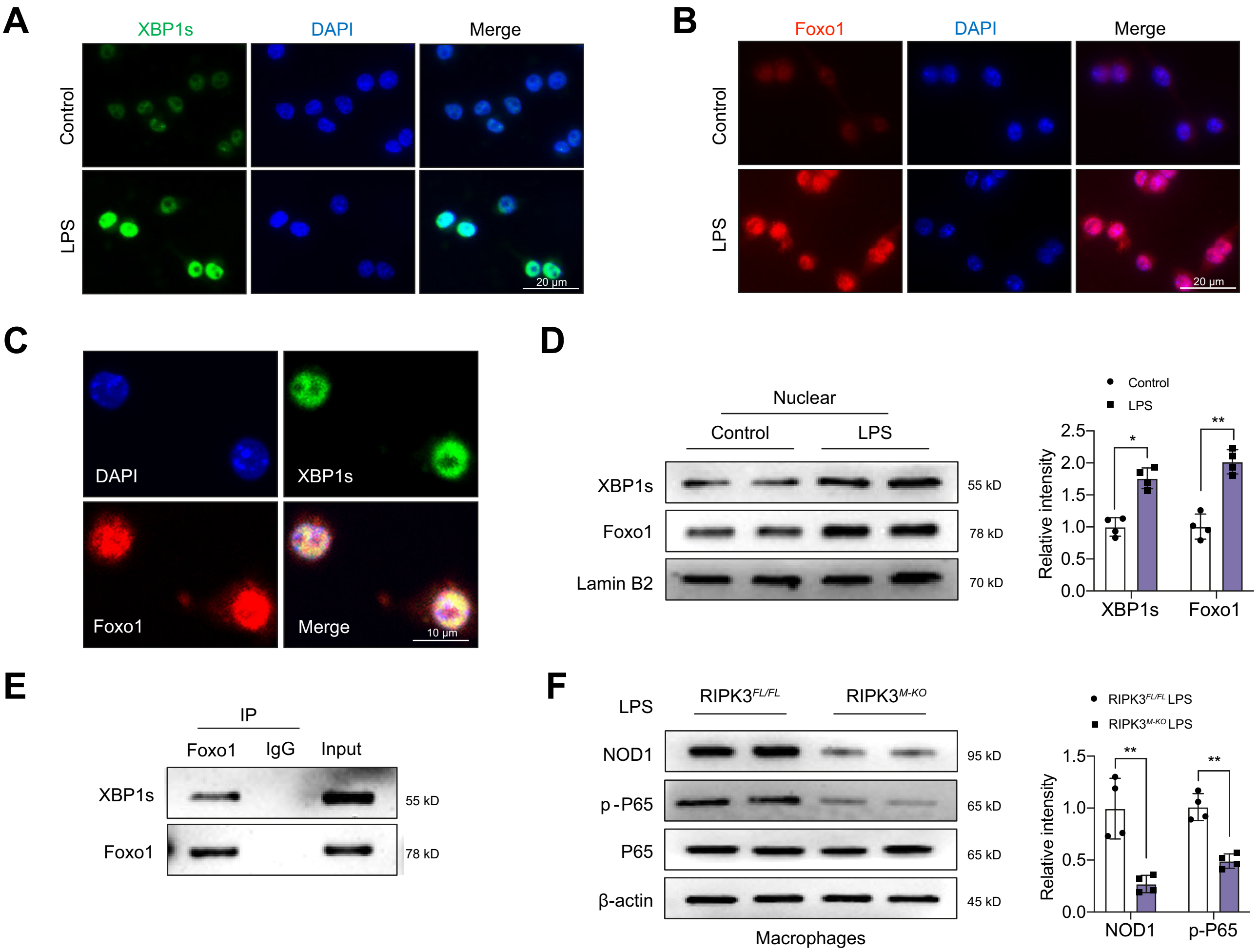

Fig. 4. XBP1 interacts with Foxo1 and regulates NOD1 activation in macrophages. BMMs (1 × 10^6) were cultured with LPS (100 ng/ml) for 6 h. (A) (B) Immunofluorescence staining for XBP1s and Foxo1 expression in macrophages after LPS stimulation (n = 4 samples/group). DAPI was used to visualise nuclei. Scale bars, 20 μm. (C) Immunofluorescence staining for macrophage XBP1s (green) and Foxo1 (red) colocalisation in LPS-stimulated macrophages. DAPI was used to visualise nuclei (blue). Scale bars, 10 μm. (D) Western blot analysis of nuclear XBP1s and Foxo1 protein expression in LPS-stimulated macrophages. (E) IP analysis of XBP1s and Foxo1 in LPS-stimulated macrophages (n = 4 samples/group). (F) BMMs from the RIPK3$^{FL/FL}$ and RIPK3^{M-KO} mice were cultured with LPS (100 ng/ml) for 6 h. Western-assisted analysis and relative density ratio of NOD1, p-P65, and P65 in LPS-stimulated macrophages. All immunoblots represent four experiments, and the data represent the mean ± SD. Statistical analysis was performed using a permutation *t* test. **p* <0.05, ***p* <0.01. BMM, bone marrow-derived macrophage; Foxo1, forkhead box O1; IP, immunoprecipitation; LPS, lipopolysaccharide; NOD1, nucleotide-binding oligomerisation domain-containing protein 1; XBP1, x-box binding protein 1; *XBP1s*, spliced XBP1.

RIPK3$^{FL/FL}$ controls. Unlike in Foxo1$^{FL/FL}$ controls, Foxo1^{M-KO} inhibited Zc3h15 and NOD1 activation (Fig. 5E), with reduced pro-inflammatory TNF-α, IL-1β, IL-6, C–X–C motif chemokine ligand 2 (CXCL-2), and CXCL-10 in LPS-stimulated macrophages (Fig. 5F). Collectively, these data indicate the critical roles of the XBP1–Foxo1 axis and its target gene Zc3h15 in the modulation of NOD1-driven inflammatory responses.

XBP1 is required for the Foxo1-targeted Zc3h15 activation and NOD1 function in macrophages

To elucidate the mechanistic role of the XBP1 in RIPK3-mediated immune regulation, we used a clustered regularly interspaced short palindromic repeats (CRISPR)/CRISPR-associated protein 9 (Cas9)-mediated XBP1 KO or activation approach. Immunofluorescence staining revealed that Foxo1-targeted Zc3h15 expression was reduced in LPS-stimulated RIPK3$^{FL/FL}$ macrophages after transfection with CRISPR/Cas9-mediated XBP1 KO vector (Fig. 6A). CRISPR/Cas9-mediated XBP1 KO inhibited the protein expression of NOD1 and p-P65 in RIPK3$^{FL/FL}$ macrophages after LPS stimulation (Fig. 6B). However, activation of XBP1 augme-nted Zc3h15 expression in LPS-stimulated RIPK3^{M-KO} macrophages, evidenced by immunofluorescence staining, which showed that CRISPR/Cas9-mediated XBP1 activation increased Zc3h15 expression (Fig. 6C), with enhanced NOD1 and P-65 activation (Fig. 6D) in RIPK3-deficient macrophages after LPS stimulation. Interestingly, the Zc3h15 expression was significantly increased in Foxo1$^{FL/FL}$ macrophages but not in Foxo1^{M-KO} macrophages after LPS stimulation (Fig. 6E and F), suggesting that the XBP1 is a key coactivator in mediating Foxo1-targeted Zc3h15 activation in RIPK3-mediated immune and inflammatory response.

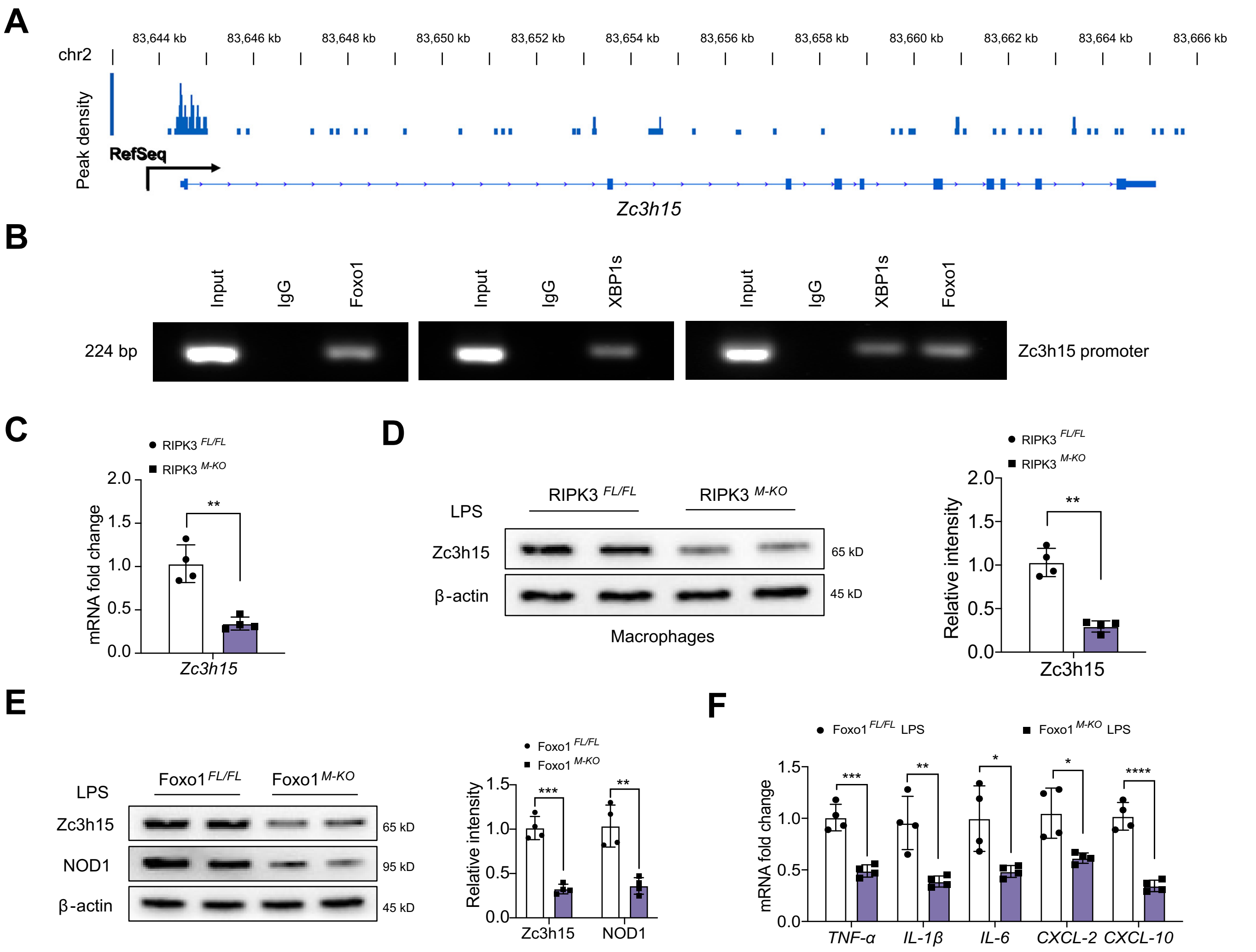

Fig. 5. The XBP1–Foxo1 axis targets Zc3h15 and modulates NOD1-driven inflammatory response in macrophages. BMMs were collected and fixed after incubating LPS (100 ng/ml). Following chromatin shearing and Foxo1 antibody selection, the precipitated DNA fragments bound by Foxo1-containing protein complexes were used for sequencing. (A) Localisation of Foxo1-binding sites on the mouse *Zc3h15* gene. The 10 exons, 9 introns, 3′ UTR, 5′ UTR, and TSSs of the mouse *Zc3h15* gene on chromosome 2 are shown. (B) ChIP-PCR analysis of Foxo1 and XBP1s binding to the *Zc3h15* promoter. Protein-bound chromatin was prepared from BMMs and immunoprecipitated with Foxo1 or XBP1s antibodies. For sequential ChIP, the protein-bound chromatin was first immunoprecipitated with the Foxo1 antibody, followed by elution with a second immunoprecipitation using XBP1s antibody. Then, the immunoprecipitated DNA was analysed by PCR. The normal IgG was used as a negative control. (C) Analysis of Zc3h15 mRNA levels in LPS-stimulated macrophages from the RIPK3$^{FL/FL}$ and RIPK3^{M-KO} mice. (n = 4 samples/group). (D) Western blot analysis and relative density ratio of Zc3h15 in LPS-stimulated macrophages from the RIPK3$^{FL/FL}$ and RIPK3^{M-KO} mice. (E) Western blot analysis and relative density ratio of Zc3h15 and NOD1 in LPS-stimulated macrophages from the Foxo1$^{FL/FL}$ and Foxo1^{M-KO} mice. (F) qRT-PCR analysis of TNF-α, IL-1β, IL-6, CXCL-2, and CXCL-10 in LPS-stimulated macrophages from the Foxo1$^{FL/FL}$ and Foxo1^{M-KO} mice. (n = 4 samples/group). All immunoblots represent four experiments, and the data represent the mean ± SD. Statistical analysis was performed using a permutation *t* test. $^{*}p$ <0.05, $^{**}p$ <0.01, $^{***}p$ <0.001, $^{****}p$ <0.001. BMM, bone marrow-derived macrophage; ChIP, chromatin immunoprecipitation; CXCL-2, C–X–C motif chemokine ligand 2; CXCL-10, C–X–C motif chemokine ligand 10; Foxo1, forkhead box O1; LPS, lipopolysaccharide; NOD1, nucleotide-binding oligomerisation domain-containing protein 1; quantitative reverse transcription PCR; RIPK3, receptor-interacting serine/threonine-protein kinase 3; TNF-α, tumour necrosis factor α; TSS, transcription start site; UTR, untranslated region; XBP1, x-box binding protein 1; *XBP1s*, spliced XBP1; *Zc3h15*, zinc finger CCCH domain-containing protein 15.

Zc3h15 is crucial to regulate NOD1-driven inflammatory response and calcineurin/TRPM7-induced cell death in response to oxidative stress

To further test the functional role of Zc3h15 in regulating macrophage NOD1 function and hepatocyte calcineurin/TRPM7 activation under cell stress conditions, we used a macrophage/hepatocyte coculture system. BMMs from the RIPK3$^{FL/FL}$ mice were transfected with CRISPR/Cas9-mediated Zc3h15 KO or control vector followed by LPS stimulation and then cocultured with primary hepatocytes supplemented with H_2O_2. Indeed, immunofluorescence staining showed that disruption of Zc3h15 reduced NOD1 expression in LPS-stimulated RIPK3$^{FL/FL}$ macrophages compared with the control vector-treated cells (Fig. 7A). Unlike in control cells, Zc3h15 deletion diminished the protein expression of NOD1 and p-P65 (Fig. 7B), with reduced mRNA levels coding for TNF-α, IL-1β, IL-6, CXCL-2, and CXCL-10 (Fig. 7C) in Zc3h15-deficient macrophages after LPS stimulation. Interestingly, increased Zc3h15 release was observed in the supernatant

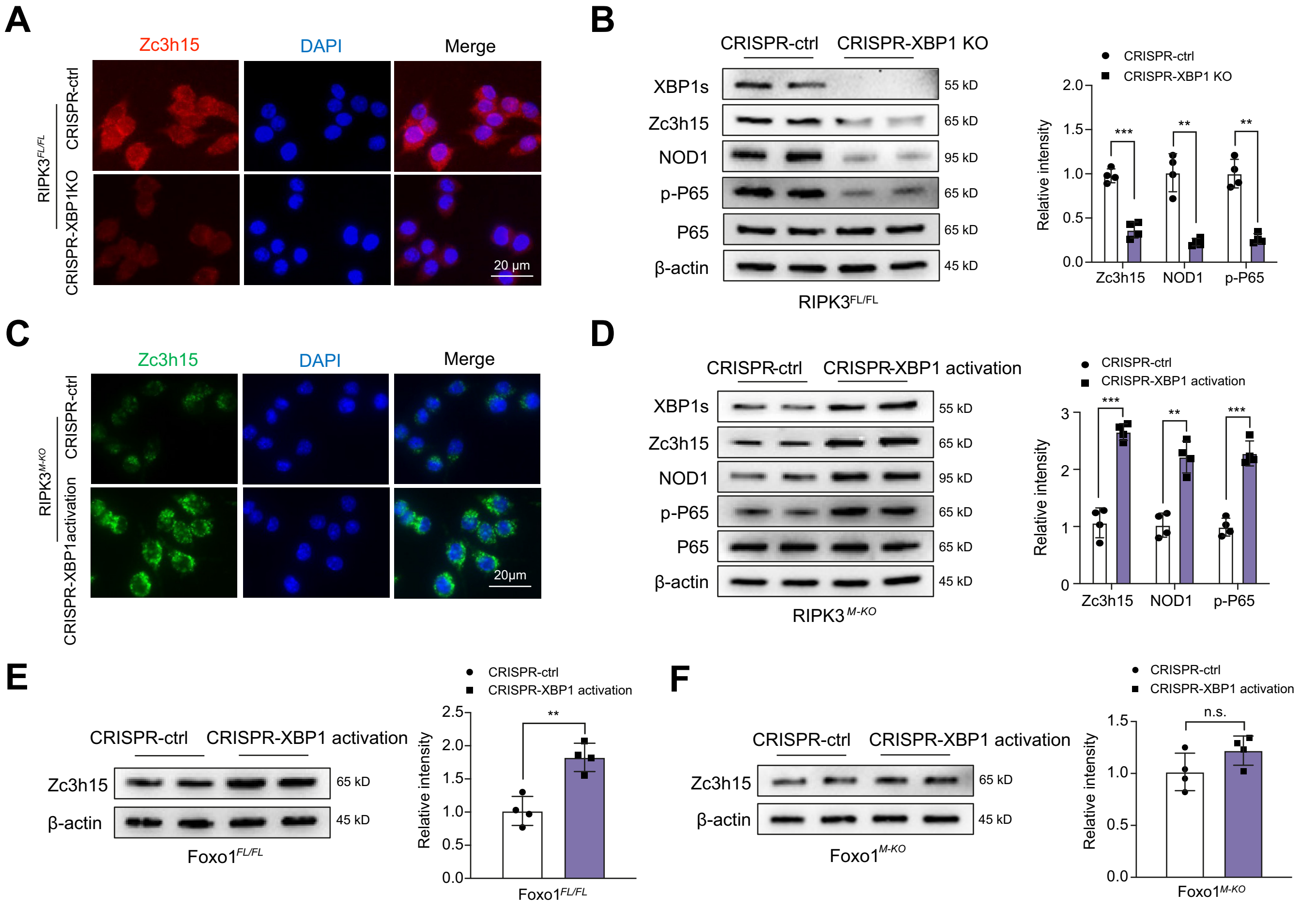

Fig. 6. XBP1 is required for the Foxo1-targeted Zc3h15 activation and NOD1 function in macrophages. BMMs from the RIPK3$^{FL/FL}$, RIPK3$^{M\text{-}KO}$, Foxo1$^{FL/FL}$, and Foxo1$^{M\text{-}KO}$ mice were transfected with p-CRISPR-XBP1 KO, p-CRISPR-XBP1 KO activation or control vector followed by 6 h of LPS (100 ng/ml) stimulation. (A) Immunofluorescence staining for the Zc3h15 expression in LPS-stimulated RIPK3$^{FL/FL}$ macrophages after transfecting p-CRISPR-XBP1 KO or control vector (n = 4 samples/group). DAPI was used to visualise nuclei. Scale bars, 20 μm. (B) Western blot analysis and relative density ratio of XBP1s, Zc3h15, NOD1, p-P65, and P65 in LPS-stimulated RIPK3$^{FL/FL}$ macrophages. (C) Immunofluorescence staining for the Zc3h15 expression in LPS-stimulated RIPK3$^{M\text{-}KO}$ macrophages after transfecting p-CRISPR-XBP1 activation or control vector (n = 4 samples/group). DAPI was used to visualise nuclei. Scale bars, 20 μm. (D) Western blot analysis and relative density ratio of XBP1s, Zc3h15, NOD1, p-P65, and P65 in LPS-stimulated RIPK3$^{M\text{-}KO}$ macrophages. (E) Western blot analysis and relative density ratio of Zc3h15 in LPS-stimulated Foxo1$^{FL/FL}$ macrophages. (F) Western blot analysis and relative density ratio of Zc3h15 in LPS-stimulated Foxo1$^{M\text{-}KO}$ macrophages. All immunoblots represent four experiments, and the data represent the mean ± SD. Statistical analysis was performed using a permutation *t* test. $^{**}p$ <0.01, $^{***}p$ <0.005. BMM, bone marrow-derived macrophage; CRISPR, clustered regularly interspaced short palindromic repeats; Foxo1, forkhead box O1; LPS, lipopolysaccharide; NOD1, nucleotide-binding oligomerisation domain-containing protein 1; RIPK3, receptor-interacting serine/threonine-protein kinase 3; XBP1, x-box binding protein 1; Zc3h15, zinc finger CCCH domain-containing protein 15.

after coculture of LPS-stimulated RIPK3$^{FL/FL}$ BMMs with H_2O_2-stressed hepatocytes (Fig. S4A). The coculture of LPS-stimulated BMMs with H_2O_2-stressed hepatocytes markedly increased the release of TRPM7 compared with those in hepatocytes exposed to H_2O_2 alone or exposed to H_2O_2 plus cocultured BMM without LPS stimulation (Fig. S4C), suggesting that LPS-stimulated macrophages with Zc3h15 release are critical in mediating TRPM7 activation in H_2O_2-stressed hepatocytes after coculture. Strikingly, LPS-stimulated Zc3h15-deficient macrophages displayed reduced hepatocyte calcineurin A and TRPM7 expression in hepatocytes with or without H_2O_2 treatment after coculture (Fig. 7D and Fig. S7A). This result was confirmed by immunofluorescence staining, which showed that disruption of macrophage Zc3h15 reduced hepatocyte TRPM7 expression after coculture (Fig. 7E). Moreover, CRISPR/Cas9-mediated Zc3h15 KO decreased TNF-α release from coculture supernatant (Fig. 7F). Notably, unlike in the control groups, LPS-stimulated Zc3h15-deficient macrophage showed reduced ROS production (Fig. 7G) and LDH release (Fig. 7H) in H_2O_2-stressed hepatocyte after coculture. Collectively, these results indicate that macrophage Zc3h15 is a critical regulator in activating NOD1-driven inflammatory response and Calcineurin/TRPM7-induced cell death in response to oxidative stress.

Adoptive transfer of Zc3h15-expressing macrophages exacerbates IR-triggered liver inflammation and hepatocyte death

Having demonstrated the importance of Zc3h15 in RIPK3-mediated immune regulation and inflammatory response in macrophages, we then examined whether Zc3h15 influenced

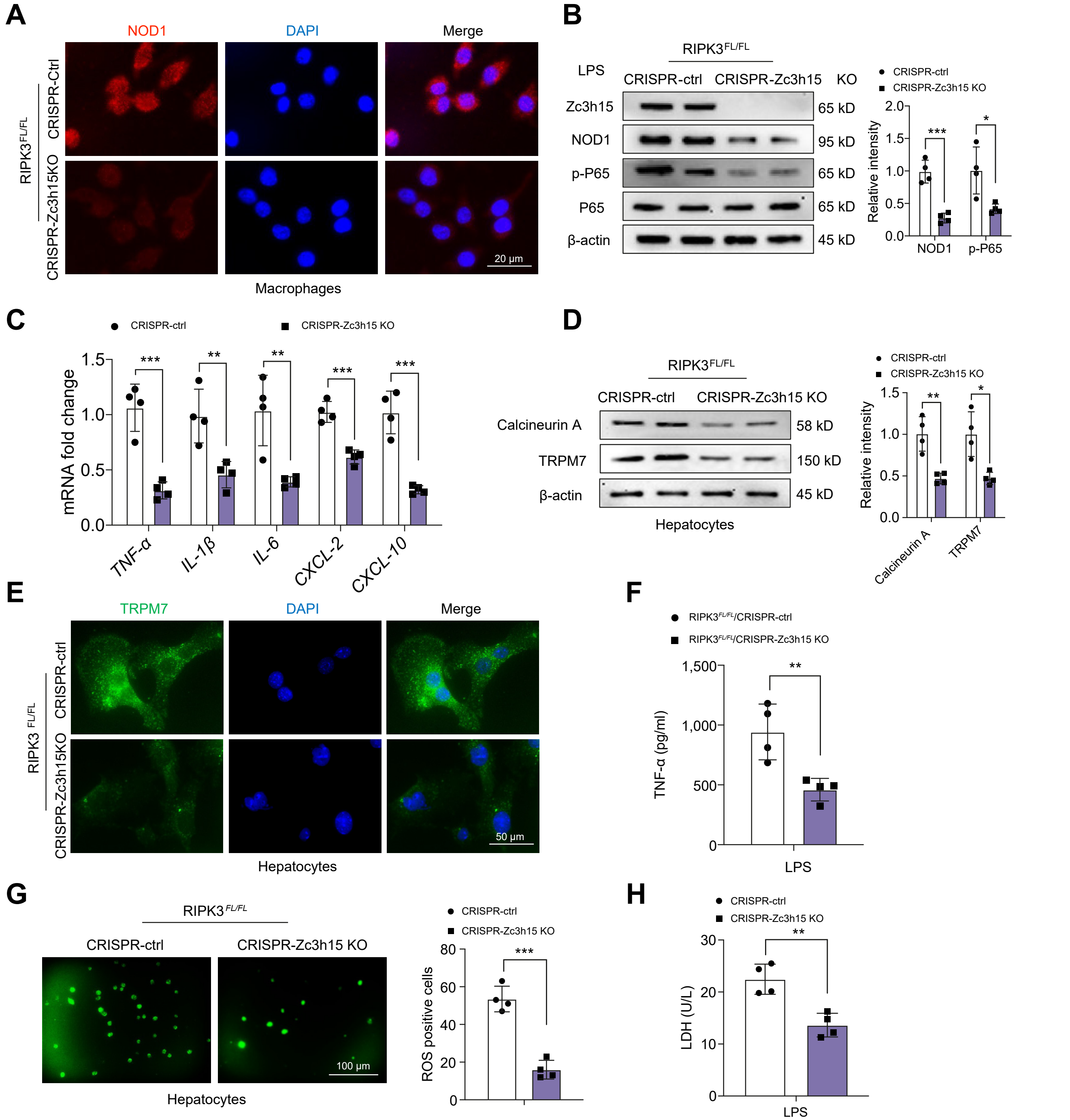

Fig. 7. Zc3h15 is crucial to regulate NOD1-driven inflammatory response and Calcineurin/TRPM7-induced cell death in response to oxidative stress. (A) BMMs from RIPK3$^{FL/FL}$ mice were transfected with p-CRISPR-Zc3h15 KO or control vector followed by 6 h of LPS (100 ng/ml) stimulation. Immunofluorescence staining for NOD1 expression in macrophages (n = 4 samples/group). DAPI was used to visualise nuclei. Scale bars, 20 μm. (B) Western blot analysis and relative density ratio of Zc3h15, NOD1, p-P65, and P65 in LPS-stimulated RIPK3$^{FL/FL}$ macrophages. (C) qRT-PCR analysis of TNF-α, IL-1β, IL-6, CXCL-2, and CXCL-10 in LPS-stimulated macrophages from the RIPK3$^{FL/FL}$ mice (n = 4 samples/group). (D) BMMs from RIPK3$^{FL/FL}$ mice were transfected with p-CRISPR-Zc3h15 KO or control vector followed by LPS stimulation and then cocultured with primary hepatocytes that were supplemented with H_2O_2 for 24 h. Western blot analysis and relative density ratio of calcineurin A and TRPM7 in H_2O_2-treated hepatocytes. (E) Immunofluorescence staining for TRPM7 expression in H2O2-treated hepatocytes (n = 4 samples/group). DAPI was used to visualise nuclei. Scale bars, 50 μm. (F) ELISA analysis of TNF-α levels in the coculture supernatant (n = 4 samples/group). (G) Detection of ROS production by carboxy-H2DFFDA in H_2O_2-treated hepatocytes from the RIPK3$^{FL/FL}$ mice. Quantification of ROS-producing hepatocytes (green) (n = 4 samples/group). Scale bars, 100 μm. (H) LDH release from the H_2O_2-treated hepatocytes in cocultures (n = 4 samples/group). All immunoblots represent four experiments, and the data represent the mean ± SD. Statistical analysis was performed using a permutation *t* test. **p* <0.05, ***p* <0.01, ****p* <0.005. BMM, bone marrow-derived macrophage; CXCL-2, C–X–C motif chemokine ligand 2; CXCL-10, C–X–C motif chemokine ligand 10; CRISPR, clustered regularly interspaced short palindromic repeats; KO, knockout; LDH, lactate dehydrogenase; LPS, lipopolysaccharide; NOD1, nucleotide-binding oligomerisation domain-containing protein 1; qRT-PCR, quantitative reverse transcription ROS, reactive oxygen species; PCR; RIPK3, receptor-interacting serine/threonine-protein kinase 3; TNF-α, tumour necrosis factor α; TRPM7, transient receptor potential cation channel subfamily M member 7; XBP1, x-box binding protein 1; Zc3h15, zinc finger CCCH domain-containing protein 15.

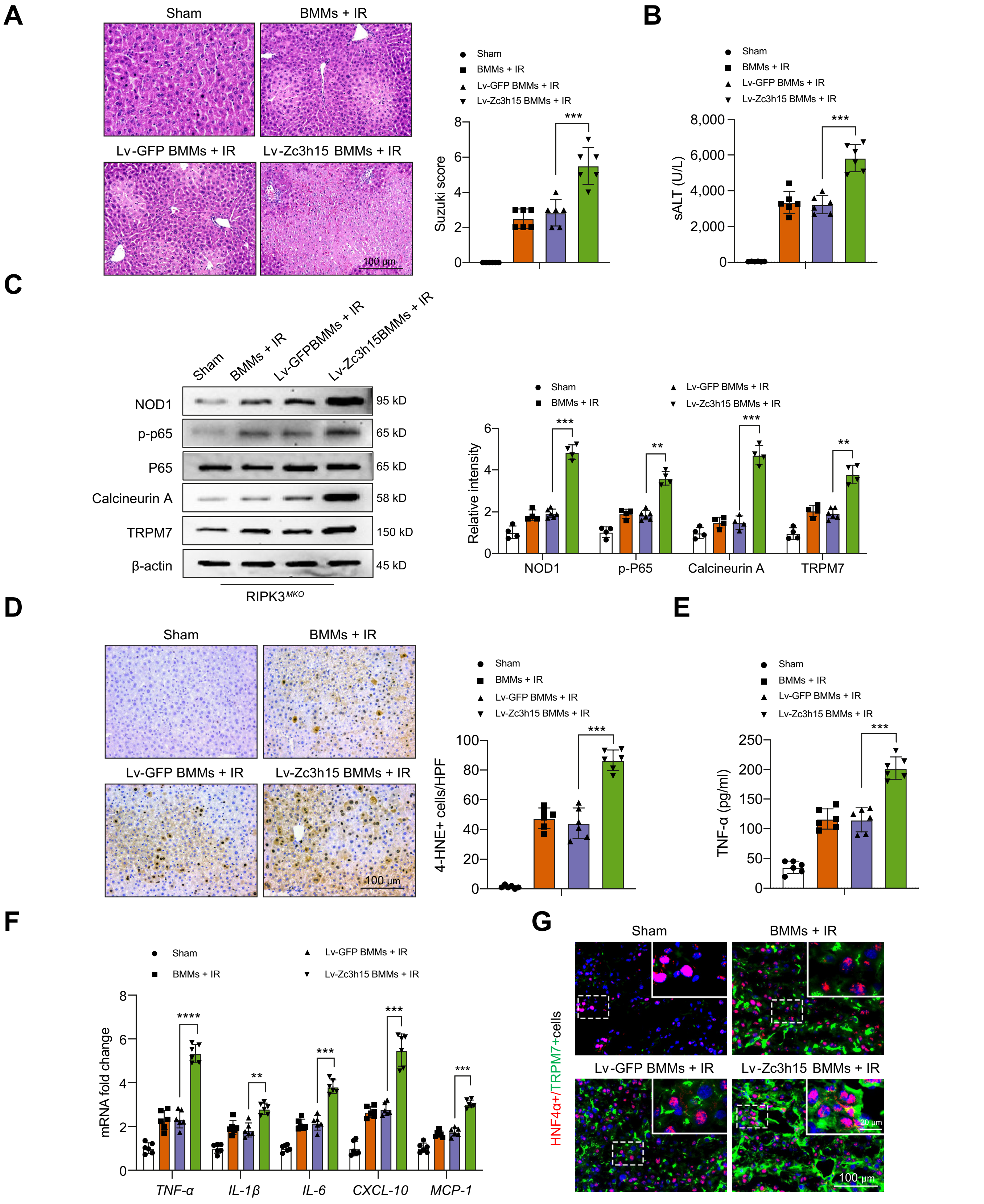

Fig. 8. Adoptive transfer of Zc3h15-expressing macrophages exacerbates IR-triggered liver inflammation and cell death. The RIPK3^{M-KO} mice were injected via tail vein with BMMs (1 × 10^6 cells/mouse) transfected with lentiviral-expressing Zc3h15 (Lv-Zc3h15) or GFP control (Lv-GFP) 24 h before ischaemia. (A) Representative histological staining (H&E) of ischaemic liver tissue (n = 6 mice/group) and Suzuki's histological score. Scale bars, 100 μm. (B) sALT levels (IU/L) (n = 6 samples/group). (C) Western-assisted analysis and relative density ratio of NOD1, p-P65, P65, calcineurin A, and TRPM7 in RIPK3^{M-KO} livers after adoptive

NOD1 and calcineurin/TRPM7 function in IR-stressed livers. BMMs were transfected with lentivirus-expressing Zc3h15 (Lv-Zc3h15) or GFP control (Lv-GFP) and then adoptively transferred into RIPK3^{M-KO} mice. The induction of Zc3h15 *in vitro* and *in vivo* was confirmed by immunohistochemistry staining, quantitative reverse-transcription (qRT)-PCR, and Western blot assay (Fig. S3). We found that Lv-Zc3h15 BMM treatment exacerbated IR-induced liver damage, as evidenced by increased Suzuki's histological score (Fig. 8A) and sALT levels (Fig. 8B), compared with that in the Lv-GFP-treated control cells. Livers treated with Lv-Zc3h15 BMMs augmented the expression of NOD1, p-P65, calcineurin A, and TRPM7 in IR-stressed livers (Fig. 8C). Moreover, overexpression of Zc3h15 increased cellular 4-hydroxynonenal (4-HNE) (Fig. 8D), a marker of ROS production[26] in Lv-Zc3h15 BMMs-treated ischaemic livers. The serum TNF-α levels were significantly increased in Lv-Zc3h15-treated groups but not in Lv-GFP-treated groups (Fig. 8E). Consistent with this result, Lv-Zc3h15 BMM treatment significantly increased the mRNA levels coding for TNF-α, IL-1β, IL-6, CXCL-10, and MCP-1 in ischaemic livers (Fig. 8F). Notably, unlike in Lv-GFP-treated controls, adoptive transfer of Lv-Zc3h15 BMMs enhanced hepatocyte TRPM7 activation as indicated by immunofluorescence staining, which showed increased hepatocyte TRPM7 expression in IR-stressed livers (Fig. 8G). Therefore, these results demonstrate the essential role of Zc3h15 for the macrophage RIPK3-mediated regulation of NOD1 function and calcineurin/TRPM7-induced hepatocyte death in IR-triggered liver inflammation.

Discussion

This study is the first to document the key role of macrophage RIPK3 signalling in regulating NOD1-driven inflammatory response and calcineurin/TRPM7-induced cell death in IR stress-induced liver injury. There are several principal findings: (i) IR stress activates macrophage IRE1α–XBP1 pathway and Foxo1 signalling in ischaemic livers; (ii) macrophage RIPK3 promotes NOD1 activation and calcineurin/TRPM7-induced hepatocyte death by triggering the XBP1–Foxo1 axis; (iii) the XBP1–Foxo1 interaction is essential for modulating its target gene *Zc3h15* function; (iv) XBP1 functions as a transcriptional coactivator of Foxo1 in regulating NOD1 and hepatocyte calcineurin/TRPM7 activation; and (v) *Zc3h15* is crucial for NOD1-driven inflammation and calcineurin/TRPM7-induced cell death cascade. Our results highlight the importance of the macrophage RIPK3-mediated XBP1–Foxo1–Zc3h15 signalling as a critical regulator of the NOD1 and calcineurin/TRPM7 function in IR-triggered liver inflammation.

Necroptosis is a regulated inflammatory type of cell death by activating RIPK3 and its downstream necroptosis executioner MLKL.[27] RIPK3 also functions as a signalling adaptor for the inflammatory response. Loss of membrane integrity results in releasing intracellular immunogenic contents, which induces an inflammatory response,[28] suggesting that necroptosis is a key driver for RIPK3-induced inflammation. Our current study revealed that IR stress induced RIPK3 activation and promoted Foxo1 signalling, with enhanced NOD1 and NF-κB activity in ischaemic livers. Moreover, NOD1 has been linked to ER stress-induced innate immune and inflammatory responses. Indeed, IR stress activated IRE1α, an ER stress sensor. Activation of IRE1α promotes the splicing of the mRNA coding for XBP1, thereby increasing protein XBP1s, a transcription factor that regulates gene expression that is involved in immune function.[29] Notably, macrophage RIPK3 deficiency inactivated the IRE1α–XBP1 pathway and depressed NOD1 activation, indicating the distinct ability of macrophage RIPK3 in controlling the IRE1α–XBP1 pathway in ER stress-mediated immune response and inflammatory cascades during liver IRI.

Foxo1 signalling pathway regulates multiple transcriptional targets involved in various cellular processes, including cell survival, stress response, apoptosis, metabolism, and inflammation.[30] Increasing Foxo1 activity activated TLR4 or NLR family pyrin domain containing 3 (NLRP3)-driven inflammatory response and tissue injury.[1,31] Disruption of Foxo1 signalling reduced susceptibility to cell death induced by oxidative stress.[32] Under cell stress conditions, Foxo1 is regulated by JNK,[33] which induces Foxo1 intracellular localisation from the cytoplasm to the nucleus and enhances Foxo1 transcription activity.[33] Consistent with this result, we found that IR stress activated JNK, which in turn stimulated Foxo1 nuclear localisation, leading to enhanced Foxo1 transcription activity. Disruption of macrophage Foxo1 reduced IR-induced liver damage with depressed NOD1 and NF-κB activation. Moreover, our *in vivo* findings showed that RIPK3 activated the IRE1α–XBP1 pathway and augmented XBP1s nuclear translocation in response to IR stress. Thus, we speculate that nuclear localisation of endogenous Foxo1 and XBP1 may be essential for modulating NOD1 activation in IR-stressed livers.

The question arises as to how XBP1 activation and Foxo1 signalling may selectively affect the NOD1 function in RIPK3-mediated immune regulation. As a key component of the ER stress response, XBP1 is spliced by IRE1α, generating functional XBP1s, which translocates into the nucleus to activate transcriptional genes involved in the pathophysiological processes of various diseases.[34] Activating XBP1 by toll-like receptors (TLRs) induces pro-inflammatory mediators in macrophages.[35] Increased ER stress activates XBP1, which is essential for inflammatory cytokine/chemokine-induced tissue inflammation and injury.[36] Moreover, the activity of Foxo1 is tightly regulated by various stimuli under pathologic and physiologic conditions.[37] Consistent with these findings, we found that IR stress augmented nuclear XBP1s and Foxo1 protein expression in Kupffer cells from ischaemic livers, implying the pivotal roles of XBP1s and Foxo1 in stress-

transfer of Lv-Zc3h15-expressing or control cells. (D) Immunohistochemistry staining of 4-HNE$^+$ cells in IR-stressed livers (n = 6 mice/group). Quantification of 4-HNE$^+$ cells, Scale bars, 100 μm. (E) ELISA analysis of serum TNF-α levels (n = 6 samples/group). (F) qRT-PCR analysis of TNF-α, IL-1β, IL-6, CXCL-10, and MCP-1 in IR-stressed livers (n = 6 samples/group). (G) Immunofluorescence staining for TRPM7 expression in hepatocytes from the RIPK3^{M-KO} mice after adoptive transfer of Lv-Zc3h15-expressing or control cells (n = 6 samples/group). DAPI was used to visualise nuclei. Scale bars, 100 and 20 μm. All immunoblots represent four experiments, and the data represent the mean ± SD. Statistical analysis was performed using a permutation *t* test. ***p* <0.01, ****p* <0.001, *****p* <0.001. 4-HNE, 4-hydroxynonenal; BMM, bone marrow-derived macrophage; CXCL-10, C–X–C motif chemokine ligand 10; GFP, green fluorescent protein; HNF4α, hepatic nuclear factor 4α; HPF, high-power field; IR, ischaemia and reperfusion; MCP-1, monocyte chemoattractant protein 1; NOD1, nucleotide-binding oligomerisation domain-containing protein 1; qRT-PCR, quantitative reverse transcription; PCR; RIPK3, receptor-interacting serine/threonine-protein kinase 3; sALT, serum alanine aminotransferase; TNF-α, tumour necrosis factor α; TRPM7, transient receptor potential cation channel subfamily M member 7; XBP1, x-box binding protein 1; Zc3h15, zinc finger CCCH domain-containing protein 15.

induced inflammatory response. Our *in vitro* study provided further evidence revealing that macrophage XBP1s and Foxo1 colocalised in the nucleus and increased nuclear expression of XBP1s and Foxo1 in response to LPS stimulation. Notably, XBP1s interacted with Foxo1 via direct binding. The ChIP and ChIP-sequencing data further revealed that XBP1s was colocalised with Foxo1 on the promoter of Zc3h15, suggesting that Zc3h15 is a target gene of Foxo1 regulated by the XBP1–Foxo1 complex. Indeed, disruption of XBP1s diminished Zc3h15, whereas activation of XBP1s increased Zc3h15 expression. Furthermore, activation of XBP1s augmented Zc3h15 induction in Foxo1$^{FL/FL}$ macrophages but not in Foxo1$^{M\text{-}KO}$ macrophages under inflammatory conditions, indicating that XBP1s acts as a transcriptional coactivator of Foxo1 in macrophage-mediated inflammatory response.

It is interesting to note that Zc3h15 is critical in activating NOD1-driven inflammatory response in IR-stressed livers. Indeed, Zc3h15 is a highly conserved eukaryotic protein associated with cell growth, transcription, and immune response.[38] Zc3h15 can regulate NF-κB signalling and MAPK activity by interacting with tumour necrosis factor receptor-associated factor 2 (TRAF2).[38] TRAF2 is an essential adaptor protein that participates in pro-inflammatory TLR signalling in macrophages.[39] Emerging evidence suggests that several CCCH zinc finger proteins, such as Zc3h15, also function as RNA-binding proteins to induce cytokine production, immune cell activation, and immune homoeostasis by modulating mRNA degradation, phosphorylation, and translation.[40] These results suggest that Zc3h15 modulates innate immune response via multiple mechanisms. In line with these findings, we found that RIPK3$^{FL/FL}$ macrophages displayed increased expression of Zc3h15 and NOD1 under inflammatory conditions. However, disruption of macrophage Zc3h15 inhibited NOD1 and NF-κB activation, with reduced pro-inflammatory mediators. Furthermore, *in vivo* adoptive transfer of Zc3h15-expressing macrophages exacerbated IR-induced liver damage and augmented NOD1 activation and ROS production in IR-stressed livers. Thus, our findings reveal a novel role of Zc3h15 in controlling NOD1-driven inflammatory response in RIPK3-mediated immune and inflammatory regulation.

Another striking finding was that macrophage RIPK3-mediated Zc3h15 could be involved in triggering IR-induced cell death pathways. Indeed, IR stress induces TNF-α and ROS production. ROS generation contributes to TNF-α-induced cell death by inducing mitochondrial membrane permeabilisation.[41] As an ion channel and functional kinase, TRPM7 is regulated by calcineurin,[42] a calcium and calmodulin-dependent serine/threonine protein phosphatase.[43] Activation of calcineurin increases inward Ca^{2+} permeation mediated by TRPM7, leading to increased ROS production under cell stress conditions.[18] Moreover, an influx of Ca^{2+} into the cytosol also leads to mitochondrial accumulation of Ca^{2+} in response to cell stress.[44] A Ca^{2+} overload links the process of necrosis and apoptosis, which is crucial for the mitochondrial permeability transition. Increased mitochondrial Ca^{2+} and ROS generation act synergistically to produce the mitochondrial permeability transition, leading to the structural and functional collapse of mitochondria and cell death.[45] In line with these findings, we found that IR stress activated calcineurin and TRPM7, whereas disruption of RIPK3 inhibited calcineurin and TRPM7 activation in IR-stressed livers, suggesting that calcineurin-mediated TRPM7 activation is crucial in RIPK3-dependent necroptosis during liver IRI. Our *in vitro* coculture system provided further evidence showing that macrophage Zc3h15 deficiency diminished TNF-α release and ROS production and inhibited calcineurin and TRPM7 in hepatocytes after coculture. Moreover, *in vivo* adoptive transfer of Zc3h15-expressing macrophages exacerbated IR-induced liver damage with enhanced calcineurin/TRPM7 activity and ROS generation. Thus, our *in vitro* and *in vivo* findings reveal the essential role of the macrophage RIPK3-mediated Zc3h15 in modulating the calcineurin/TRPM7-induced cell death cascade in IR stress-induced liver inflammatory injury.

In conclusion, we identify a previously unrecognised role of macrophage RIPK3-mediated XBP1–Foxo1–Zc3h15 signalling in regulating NOD1-dependent inflammation and calcineurin/TRPM7-induced hepatocyte death during liver IRI. RIPK3 drives NOD1 and calcineurin-mediated TRPM7 activation by promoting the XBP1–Foxo1 axis and its target gene Zc3h15 in response to IR stress. By identifying the molecular regulatory mechanism of the macrophage RIPK3-mediated XBP1–Foxo1–Zc3h15 pathway in IR-stressed livers, our findings provide potential therapeutic targets for stress-induced liver inflammation and injury.

Abbreviations

4-HNE, 4-hydroxynonenal; BMM, bone marrow-derived macrophage; Cas9, CRISPR-associated protein 9; ChIP, chromatin immunoprecipitation; ChIP-seq, ChIP coupled with sequencing; CRISPR, clustered regularly interspaced short palindromic repeats; CXCL-2, C–X–C motif chemokine ligand 2; CXCL-10, C–X–C motif chemokine ligand 10; ER, endoplasmic reticulum; Foxo1, forkhead box O1; FRT, Flippase recognition target; IR, ischaemia and reperfusion; IRE1α, inositol-requiring transmembrane kinase/endoribonuclease 1α; IRI, ischaemia–reperfusion injury; JNK, c-Jun N-terminal kinase; LDH, lactate dehydrogenase; LPS, lipopolysaccharide; Lyz2, Lysozyme 2; MAPK, mitogen-activated protein kinase; MCP-1, monocyte chemoattractant protein 1; MLKL, mixed lineage kinase domain-like pseudokinase; neo, neomycin resistance; NOD1, nucleotide-binding oligomerisation domain-containing protein 1; qRT-PCR, quantitative reverse transcription PCR; RIPK3, receptor-interacting serine/threonine-protein kinase 3; ROS, reactive oxygen species; sALT, serum alanine aminotransferase; sAST, serum aspartate aminotransferase; TLR, Toll-like receptor; TLR4, Toll-like receptor 4; TNF, tumour necrosis factor; TNF-α, tumour necrosis factor α; TNFR1, tumour necrosis factor receptor 1; TRAF2, tumour necrosis factor receptor-associated factor 2; TRPM7, transient receptor potential cation channel subfamily M member 7; XBP1, x-box binding protein 1; XBP1s, spliced XBP1; Zc3h15, zinc finger CCCH domain-containing protein 15.

Financial support

This work was supported by NIH grants R01AI139552, P01AI120994, R21AI146742, R21AI112722, and R21AI115133.

Conflicts of interest

The authors declare no conflict of interest.

Please refer to the accompanying ICMJE disclosure forms for further details.

Authors' contributions

Performed *in vivo* and *in vitro* experiments and data analysis: XQ, TY, XW, DX, YY. Generated conditional knockout mice and data analysis: DX, LJ. Participated in scientific discussion: JL, QX, DGF. Contributed to the study concept, research design, and data analysis, and wrote the manuscript: BK.

Data availability statement

The data that support the findings of this study are available from the corresponding author upon reasonable request.

Supplementary data

Supplementary data to this article can be found online at https://doi.org/10.1016/j.jhepr.2023.100879.

References

Author names in bold designate shared co-first authorship

[1] **Li C**, **Sheng M**, Lin Y, Xu D, Tian Y, Zhan Y, et al. Functional crosstalk between myeloid Foxo1-beta-catenin axis and Hedgehog/Gli1 signaling in oxidative stress response. Cell Death Differ 2021;28:1705–1719.

[2] **Wu L**, **Xiong X**, Wu X, Ye Y, Jian Z, Zhi Z, et al. Targeting oxidative stress and inflammation to prevent ischemia–reperfusion injury. Front Mol Neurosci 2020;13:28.

[3] **Yue S**, **Zhu J**, Zhang M, Li C, Zhou X, Zhou M, et al. The myeloid heat shock transcription factor 1/β-catenin axis regulates NLR family, pyrin domain-containing 3 inflammasome activation in mouse liver ischemia/reperfusion injury. Hepatology 2016;64:1683–1698.

[4] **Lu L**, **Yue S**, Jiang L, Li C, Zhu Q, Ke M, et al. Myeloid Notch1 deficiency activates the RhoA/ROCK pathway and aggravates hepatocellular damage in mouse ischemic livers. Hepatology 2018;67:1041–1055.

[5] Chakrabarti S, Visweswariah SS. Intramacrophage ROS primes the innate immune system via JAK/STAT and Toll activation. Cell Rep 2020;33:108368.

[6] **Keestra-Gounder AM**, **Byndloss MX**, Seyffert N, Young BM, Chavez-Arroyo A, Tsai AY, et al. NOD1 and NOD2 signalling links ER stress with inflammation. Nature 2016;532:394–397.

[7] **Travassos LH**, **Carneiro LA**, Girardin SE, Boneca IG, Lemos R, Bozza MT, et al. Nod1 participates in the innate immune response to *Pseudomonas aeruginosa*. J Biol Chem 2005;280:36714–36718.

[8] **Masumoto J**, **Yang K**, Varambally S, Hasegawa M, Tomlins SA, Qiu S, et al. Nod1 acts as an intracellular receptor to stimulate chemokine production and neutrophil recruitment in vivo. J Exp Med 2006;203:203–213.

[9] Motomura Y, Kanno S, Asano K, Tanaka M, Hasegawa Y, Katagiri H, et al. Identification of pathogenic cardiac $CD11c^+$ macrophages in Nod1-mediated acute coronary arteritis. Arterioscler Thromb Vasc Biol 2015;35:1423–1433.

[10] **González-Ramos S**, **Fernández-García V**, Recalde M, Rodriguez C, Martínez-González J, Andres V, et al. Deletion or Inhibition of NOD1 favors plaque stability and attenuates atherothrombosis in advanced atherogenesis. Cells 2020;9:2067.

[11] Pei G, Zyla J, He L, Moura-Alves P, Steinle H, Saikali P, et al. Cellular stress promotes NOD1/2-dependent inflammation via the endogenous metabolite sphingosine-1-phosphate. EMBO J 2021;40:e106272.

[12] Sharma A, Singh S, Ahmad S, Gulzar F, Schertzer JD, Tamrakar AK. NOD1 activation induces oxidative stress via NOX1/4 in adipocytes. Free Radic Biol Med 2021;162:118–128.

[13] **Liu S**, **Joshi K**, Denning MF, Zhang J. RIPK3 signaling and its role in the pathogenesis of cancers. Cell Mol Life Sci 2021;78:7199–7217.

[14] Sun L, Wang H, Wang Z, He S, Chen S, Liao D, et al. Mixed lineage kinase domain-like protein mediates necrosis signaling downstream of RIP3 kinase. Cell 2012;148:213–227.

[15] Sun X, Lee J, Navas T, Baldwin DT, Stewart TA, Dixit VM. RIP3, a novel apoptosis-inducing kinase. J Biol Chem 1999;274:16871–16875.

[16] Wang H, Sun L, Su L, Rizo J, Liu L, Wang LF, et al. Mixed lineage kinase domain-like protein MLKL causes necrotic membrane disruption upon phosphorylation by RIP3. Mol Cell 2014;54:133–146.

[17] Calvo-Rodriguez M, Hou SS, Snyder AC, Kharitonova EK, Russ AN, Das S, et al. Increased mitochondrial calcium levels associated with neuronal death in a mouse model of Alzheimer's disease. Nat Commun 2020;11:2146.

[18] **Aarts M**, **Iihara K**, **Wei WL**, Xiong ZG, Arundine M, Cerwinski W, et al. A key role for TRPM7 channels in anoxic neuronal death. Cell 2003;115:863–877.

[19] Newton K. RIPK1 and RIPK3: critical regulators of inflammation and cell death. Trends Cell Biol 2015;25:347–353.

[20] **Gautheron J**, **Vucur M**, **Reisinger F**, Cardenas DV, Roderburg C, Koppe C, et al. A positive feedback loop between RIP3 and JNK controls non-alcoholic steatohepatitis. EMBO Mol Med 2014;6:1062–1074.

[21] Preston SP, Stutz MD, Allison CC, Nachbur U, Gouil Q, Tran BM, et al. Epigenetic silencing of RIPK3 in hepatocytes prevents MLKL-mediated necroptosis from contributing to liver pathologies. Gastroenterology 2022;163:1643–1657.e14.

[22] He S, Liang Y, Shao F, Wang X. Toll-like receptors activate programmed necrosis in macrophages through a receptor-interacting kinase-3-mediated pathway. Proc Natl Acad Sci U S A 2011;108:20054–20059.

[23] **Zhu P**, **Hu S**, Jin Q, Li D, Tian F, Toan S, et al. Ripk3 promotes ER stress-induced necroptosis in cardiac IR injury: a mechanism involving calcium overload/XO/ROS/mPTP pathway. Redox Biol 2018;16:157–168.

[24] Suzuki S, Toledo-Pereyra LH, Rodriguez FJ, Cejalvo D. Neutrophil infiltration as an important factor in liver ischemia and reperfusion injury. Modulating effects of FK506 and cyclosporine. Transplantation 1993;55:1265–1272.

[25] **Kaser A**, **Lee AH**, Franke A, Glickman JN, Zeissig S, Tilg H, et al. XBP1 links ER stress to intestinal inflammation and confers genetic risk for human inflammatory bowel disease. Cell 2008;134:743–756.

[26] Liou GY, Storz P. Detecting reactive oxygen species by immunohistochemistry. Methods Mol Biol 2015;1292:97–104.

[27] Choi ME, Price DR, Ryter SW, Choi AMK. Necroptosis: a crucial pathogenic mediator of human disease. JCI Insight 2019;4:128834.

[28] Moriwaki K, Chan FK. The inflammatory signal adaptor RIPK3: functions beyond necroptosis. Int Rev Cell Mol Biol 2017;328:253–275.

[29] Park SM, Kang TI, So JS. Roles of XBP1s in transcriptional regulation of target genes. Biomedicines 2021;9:791.

[30] Obsil T, Obsilova V. Structure/function relationships underlying regulation of FOXO transcription factors. Oncogene 2008;27:2263–2275.

[31] Fan W, Morinaga H, Kim JJ, Bae E, Spann NJ, Heinz S, et al. FoxO1 regulates Tlr4 inflammatory pathway signalling in macrophages. EMBO J 2010;29:4223–4236.

[32] Sengupta A, Molkentin JD, Paik JH, DePinho RA, Yutzey KE. FoxO transcription factors promote cardiomyocyte survival upon induction of oxidative stress. J Biol Chem 2011;286:7468–7478.

[33] Kawamori D, Kaneto H, Nakatani Y, Matsuoka TA, Matsuhisa M, Hori M, et al. The forkhead transcription factor Foxo1 bridges the JNK pathway and the transcription factor PDX-1 through its intracellular translocation. J Biol Chem 2006;281:1091–1098.

[34] Sha H, He Y, Yang L, Qi L. Stressed out about obesity: IRE1α-XBP1 in metabolic disorders. Trends Endocrinol Metab 2011;22:374–381.

[35] Martinon F, Chen X, Lee AH, Glimcher LH. TLR activation of the transcription factor XBP1 regulates innate immune responses in macrophages. Nat Immunol 2010;11:411–418.

[36] Gargalovic PS, Gharavi NM, Clark MJ, Pagnon J, Yang WP, He A, et al. The unfolded protein response is an important regulator of inflammatory genes in endothelial cells. Arterioscler Thromb Vasc Biol 2006;26:2490–2496.

[37] Brown AK, Webb AE. Regulation of FOXO factors in mammalian cells. Curr Top Dev Biol 2018;127:165–192.

[38] Capalbo G, Mueller-Kuller T, Koschmieder S, Klein HU, Ottmann OG, Hoelzer D, et al. Characterization of ZC3H15 as a potential TRAF-2-interacting protein implicated in the NFκB pathway and overexpressed in AML. Int J Oncol 2013;43:246–254.

[39] Jin J, Xiao Y, Hu H, Zou Q, Li Y, Gao Y, et al. Proinflammatory TLR signalling is regulated by a TRAF2-dependent proteolysis mechanism in macrophages. Nat Commun 2015;6:5930.

[40] Fu M, Blackshear PJ. RNA-binding proteins in immune regulation: a focus on CCCH zinc finger proteins. Nat Rev Immunol 2017;17:130–143.

[41] Kim JJ, Lee SB, Park JK, Yoo YD. TNF-α-induced ROS production triggering apoptosis is directly linked to Romo1 and Bcl-X_L. Cell Death Differ 2010;17:1420–1434.

[42] **Turlova E**, **Wong R**, Xu B, Li F, Du L, Habbous S, et al. TRPM7 mediates neuronal cell death upstream of calcium/calmodulin-dependent protein kinase II and calcineurin mechanism in neonatal hypoxic–ischemic brain injury. Transl Stroke Res 2021;12:164–184.

[43] Bandyopadhyay J, Lee J, Bandyopadhyay A. Regulation of calcineurin, a calcium/calmodulin-dependent protein phosphatase, in *C. elegans*. Mol Cells 2004;18:10–16.

[44] Duchen MR. Mitochondria and calcium: from cell signalling to cell death. J Physiol 2000;529 Pt1:57–68.

[45] Lemasters JJ, Theruvath TP, Zhong Z, Nieminen AL. Mitochondrial calcium and the permeability transition in cell death. Biochim Biophys Acta 2009;1787:1395–1401.

Journal of Hepatology, Volume *5*

Supplemental information

Macrophage RIPK3 triggers inflammation and cell death via the XBP1–Foxo1 axis in liver ischaemia–reperfusion injury

Xiaoye Qu, Tao Yang, Xiao Wang, Dongwei Xu, Yeping Yu, Jun Li, Longfeng Jiang, Qiang Xia, Douglas G. Farmer, and Bibo Ke

Macrophage RIPK3 triggers inflammation and cell death via the XBP1–Foxo1 axis in liver ischaemia–reperfusion injury

Xiaoye Qu, Tao Yang, Xiao Wang, Dongwei Xu, Yeping Yu, Jun Li, Longfeng Jiang, Qiang Xia, Douglas G. Farmer, Bibo Ke

Table of contents

Supplementary materials and methods....................2

Fig. S1....................9

Fig. S2....................10

Fig. S3....................11

Fig. S4....................12

Fig. S5....................13

Fig. S6....................14

Fig. S7....................15

Table S1....................16

Supplementary references....................17

Supplementary materials and methods

Animals. The floxed RIPK3 ($RIPK3^{FL/FL}$) mice (B6;129-*RIPK3*$^{tm1.1Fkmc}$/J) and the mice expressing Cre recombinase under the control of the Lysozyme 2 (Lyz2) promoter (LysM-Cre) were obtained from The Jackson Laboratory (Bar Harbor, ME). The targeting vector is designed to insert a loxP site and a FRT-flanked neomycin resistance (neo) upstream of exon 10. An enhanced green fluorescent protein (EGFP) sequence, followed by a second loxP site, is inserted at the end of the coding region. Flp-mediated recombination removed the FRT-flanked neo cassette. This strain was maintained on a mixed 129 and C57BL/6 genetic background. To generate myeloid-specific RIPK3 knockout ($RIPK3^{M\text{-}KO}$) mice, a homozygous loxP-flanked RIPK3 mouse was mated with a homozygous Lyz2-Cre mouse to create the F1 mice that were heterozygous for a loxP-flanked RIPK3 allele and heterozygous for the Lyz2-Cre. The F1 mice were then backcrossed to the homozygous loxP-flanked RIPK3 mice, resulting in the generation of $RIPK3^{M\text{-}KO}$ (25% of the offspring), which were homozygous for the loxP-flanked RIPK3 allele and heterozygous for the Lyz2-Cre allele (Fig. S1). The myeloid-specific Foxo1 knockout ($Foxo1^{M\text{-}KO}$) mice were generated as described [1]. Mouse genotyping was performed using a standard protocol with primers described in the JAX Genotyping protocols database. Male mice at 6-8 weeks of age were used in all experiments. This study was performed in strict accordance with the recommendations in the *Guide for the Care and Use of Laboratory Animals* published by the National Institutes of Health. Animal protocols were approved by the Institutional Animal Care and Use Committee of The University of California at Los Angeles.

Mouse liver IRI model. We used an established mouse model of warm hepatic ischemia (90min) followed by reperfusion (6h) [2]. Mice were injected with heparin (100U/kg), and an atraumatic clip was used to interrupt the arterial/portal venous blood supply to the cephalad liver lobes. After 90min of ischemia, the clip was removed, and mice were sacrificed at 6h of reperfusion. Some animals were injected via tail vein with Zc3h15-expressing bone marrow-

derived macrophages (BMMs) or control cells ($1x10^6$ cells in 0.1 ml of PBS/mouse) 24h before ischemia.

Hepatocellular function assay. Serum alanine aminotransferase (sALT) levels, an indicator of hepatocellular injury, were measured by ALT and AST kit (ThermoFisher, Waltham, MA) according to the manufacturer's instructions.

Histology, immunohistochemistry, and immunofluorescence staining. Liver sections (5-μm) were stained with hematoxylin and eosin (H&E). The severity of IRI was graded using Suzuki's criteria [3]. Liver macrophages were detected using primary rat $CD11b^+$ monoclonal antibodies (mAb) (Abcam, Cambridge, MA) and secondary AlexFluor488-conjugated AffiniPure donkey anti-rat IgG (Jackson Immunoresearch, West Grove, PA) for immunofluorescence staining. DAPI was used for nuclear counterstaining. Liver neutrophils were detected by immunohistochemistry (IHC) or Immunofluorescence staining using primary rat Ly6G mAb (ThermoFisher Scientific). Immunofluorescence staining of RIPK3 in Kupffer cells or TRMP7 in hepatocytes were analyzed in the liver sections using primary rabbit RIPK3 mAb (Cell Signaling Technology, Danvers, MA) and rat CD68 mAb (Bio-Rad, Hercules, CA) or mouse TRMP7 mAb (Santa Cruz Biotechnology) and rabbit HNF4α mAb (Abcam). The gene expression was detected in liver sections by Immunofluorescence and immunohistochemistry staining using primary rabbit Zc3h15 (ThermoFisher Scientific) and mouse NOD1 (Santa Cruz Biotechnology) Abs. The primary rabbit XBP1s (Cell Signaling Technology) and mouse Foxo1 (Santa Cruz Biotechnology) Abs, the secondary AlexFluor488-conjugated AffiniPure donkey anti-rabbit IgG Ab, Cy5-conjugated AffiniPure donkey anti-mouse IgG Ab (Jackson Immunoresearch) were used for staining XBP1s and Foxo1 positive cells according to the manufacturer's instructions. Images for immunofluorescence staining were captured using a fluorescence microscope (Keyence BZ-X810, Osaka, Japan) and analyzed using Image-pro Plus software. Positive cells were counted blindly in 10 HPF/section (x200).

Quantitative RT-PCR analysis. Total RNA was purified from liver tissue or cell cultures using RNeasy Mini Kit (Qiagen, Chatsworth, CA) according to the manufacturer's instructions. Reverse transcription to cDNA was performed by using SuperScript III First-Strand Synthesis System (ThermoFisher Scientific). Quantitative real-time PCR was carried out using the QuantStudio 3 (Applied Biosystems by ThermoFisher Scientific). In a final reaction volume of 25μl, the following were added: 1× SuperMix (Platinum SYBR Green qPCR Kit; Invitrogen) cDNA and 10μM of each primer. Amplification conditions were: 50°C (2min), 95°C (5min), followed by 40 cycles of 95°C (15sec) and 60°C (30sec). The primer sequences that amplify TNF-α, IL-1β, IL-6, CXCL-2, CXCL10, MCP-1, Zc3h15, and HPRT were shown in Supplementary Table 1. The target gene expressions were calculated by their ratios to the housekeeping gene HPRT.

Western blot analysis. Protein was extracted from liver tissue or cell cultures with ice-cold protein lysis buffer (50mM Tris, 150mM NaCl, 0.1% sodium dodecyl sulfate, 1% sodium deoxycholate, 1% Triton-100). The buffer contains 1% proteinase and phosphatase inhibitor cocktails (Sigma-Aldrich, St. Louis, MO). Proteins (30 μg/sample) in SDS-loading buffer (50mM Tris, pH 7.6, 10% glycerol, 1% SDS) were subjected to 4-20% SDS-polyacrylamide gel electrophoresis (PAGE) and transferred to nitrocellulose membrane (Bio-Rad). The membrane was blocked with 5% dry milk and 0.1% Tween 20 (USB, Cleveland, OH). The nuclear and cytosolic fractions were prepared with NE-PER Nuclear and Cytoplasmic Extraction Reagents (ThermoFisher Scientific). The RIPK3, IRE1α, NOD1, RIP2, p-P65, P-65, p-JNK, JNK, Foxo1, XBP1s, Lamin B2, and β-actin (Cell Signaling Technology), Calcineurin A and Zc3h15 (ThermoFisher Scientific), and TRPM7 (Santa Cruz Biotechnology) were used. The membranes were incubated with Abs and then added Western ECL substrate mixture (Bio-Rad) for imaging with the iBright FL1000 (ThermoFisher Scientific). Relative quantities of protein were determined by comparing the β-actin expression using iBright image analysis software (ThermoFisher Scientific).

Isolation of primary hepatocytes, Kupffer cells, and bone marrow-derived macrophages. Primary hepatocytes, Kupffer cells, and BMMs from the $RIPK3^{FL/FL}$, $RIPK3^{M\text{-}KO}$, or wild-type (WT) mice were isolated as described [2]. In brief, livers were perfused in situ with warmed (37°C) HBSS solution, followed by a collagenase buffer (collagenase type IV, Sigma-Aldrich). The Perfused livers were dissected and teased through 70-μm nylon mesh cell strainers (BD Biosciences, San Jose, CA). The nonparenchymal cells (NPCs) were separated from hepatocytes by centrifuging at 50 × *g* 2min three times. The NPCs were then suspended in HBSS and layered onto a 50%/25% two-step Percoll gradient (Sigma) in a 50-ml conical centrifuge tube and centrifuged at 1800 × *g* at 4°C for 15min. The Kupffer cells in the middle layer were collected and plated to cell culture dishes in DMEM with 10% FBS, 10mM HEPES, 2mM GlutaMax, 100 U/ml penicillin, and 100 μg/ml streptomycin for 15min at 37°C. Murine bone-derived macrophages (BMMs) were generated as described [2]. In brief, bone marrow cells were removed from the femurs and tibias of the $RIPK3^{FL/FL}$, $RIPK3^{M\text{-}KO}$, or WT mice and cultured in DMEM supplemented with 10% FCS and 15% L929-conditioned medium for seven days.

Flow cytometry analysis. $1X10^5$ primary hepatocytes or liver macrophages (Kupffer cells) were washed with staining medium (phosphate-buffered saline containing 3% fetal bovine serum), and then incubated with fluorescence-conjugated antibodies. We used the Alexa Fluor 488-conjugated anti-ASGR1 polyclonal antibody (ThermoFisher Scientific) to detect hepatocytes, and Alexa Fluor 488 mouse IgG1k isotype control (BD Biosciences) was used. The BD Horizon PE-CF594 rat anti-mouse F4/80 (BD Biosciences) was used for detecting macrophages, and the PE-conjugated mouse IgG1k isotype control (BD Biosciences) was also used. The fluorescence-labeled cells were run through a flow cytometer (LSRFortessaX-20, BD Biosciences). All data were analyzed with FlowJo software (Tree Star, Inc.)

Co-culture of macrophages and primary hepatocytes. Primary hepatocytes were cultured in 6-well plates at a concentration of $4x10^5$ cells per well. After 24h, the 0.4μm-pore size

transwell inserts (Corning) containing $1x10^6$ BMMs were placed into the 6-well plate with the initially seeded hepatocytes. The co-cultures were incubated for 12h with or without adding H_2O_2 (200 μM) in the lower chamber.

ELISA assay. Cell culture supernatants were harvested for cytokine analysis. ELISA kits were used to measure the Zc3h15 (Biohippo, Gaithersburg, MD), RIPK3 (MyBioSource, San Diego, CA), TRPM7 (MyBioSource), and TNF-α (ThermoFisher Scientific) levels according to the manufacturer's instructions.

LDH activity assay. BMMs ($1x10^6$) were cultured with primary hepatocytes ($4x10^5$/well) for 12h with or without adding H_2O_2 (200 μM) in the lower chamber. The activity of lactate dehydrogenase (LDH) in the cell culture medium from the lower chamber was measured with a commercial LDH activity assay kit (Stanbio Laboratory, Boerne, TX) according to manufacturer's instructions.

Reactive oxygen species assay. ROS production in Kupffer cells was measured using the 5-(and-6)-carboxy-2',7'-difluorodihydrofluorescein diacetate (Carboxy-H2DFFDA, ThermoFisher Scientific), as described [2]. In brief, Kupffer cells ($2x10^5$) were isolated from ischemic livers and cultured on collagen-coated cover slips without or with LPS (100ng/ml) for 2h at 37°C. After washing with PBS, cells were incubated with 10μM of Carboxy-H2DFFDA. The Carboxy-H2DFFDA was converted to a green-fluorescent form when hydrolyzed by intracellular esterase and oxidized in the cells. Cells were fixed with 2% paraformaldehyde and stained with Hoechst dye. ROS produced by Kupffer cells were analyzed and quantified by fluorescence microscopy. Positive green fluorescent-labeled cells were counted blindly in 10 HPF/section (x200).

In vitro transfection. BMMs ($1x10^6$/well) were cultured for seven days and then and then transfected with CRISPR/Cas9-XBP1 knockout (KO), CRISPR-XBP1 activation, CRISPR/Cas9-

Zc3h15 KO, or control vector (Santa Cruz Biotechnology) by using Lipofectamine™ 3000 according to the manufacturer's instructions (ThermoFisher Scientific). After 24-48h, cells were supplemented with LPS (100 ng/ml) for an additional 6h. For lentivirus-mediated gene transfer, BMMs ($1x10^6$/well) were added with lentivirus-mediated Zc3h15 (Lv-Zc3h15) (Santa Cruz Biotechnology) or Lv-GFP control (at a multiplicity of infection 10) (Applied Biological Materials, Richmond, BC, Canada) and incubated at 37°C overnight. The medium was removed and replaced with fresh medium. After 48h, cells were harvested for *in vivo* adoptive transfer.

Immunoprecipitation analysis. BMMs after LPS stimulation were lysed in NP-40 lysis buffer (50mM Tris pH7.4, 10 mM EDTA, 150 mM NaCl, 1% NP-40, ThermoFisher Scientific) containing protease inhibitors. The lysates were incubated with XBP1s (Cell Signaling Technology), Foxo1 (Cell Signaling Technology), or control IgG and protein A/G beads at 4 ^{o}C overnight. After immunoprecipitation, the immunocomplexes were washed with lysis buffer three times and analyzed by standard immunoblot procedures.

Chromatin immunoprecipitation (ChIP). The ChIP analysis was carried out using ChIP Assay Kit (Abcam). Briefly, BMMs were treated with 1% formaldehyde for 10 min to cross-link proteins and chromatin. The reaction was stopped by adding 0.125M glycine for 5 min. Cells were washed with ice-cold PBS and then resuspended with ChIP lysis buffer for 10 min. Cell lysates were centrifuged to pellet the nuclei. The cell nuclei were resuspended in nuclei lysis buffer and then subjected to sonication for 15 min. Purified chromatin was analyzed on a 1.5 % agarose gel to analyze DNA fragment size. The sheared chromatin was immunoprecipitated with XBP1s (Cell Signaling Technology) or Foxo1 antibody (Cell Signaling Technology) overnight. As a control, the normal IgG was used as a replacement for XBP1s or Foxo1 antibody. The antibody/chromatin samples were mixed with protein A sepharose beads. Protein-DNA complexes were washed and eluted, followed by a cross-link reversal step, and the resulting DNA was purified. For sequential ChIP, sheared chromatin was first immunoprecipitated with XBP1s antibody, followed by elution

with a second immunoprecipitation using Foxo1 antibody. DNA from each immunoprecipitation reaction was examined by PCR. The primer for the Foxo1-responsive region of *Zc3h15* promoter: forward: 5'- CTGTCGCAAAGGCCACAT -3', reverse: 5'- CGATGATCTTCTCCTTCTTCTT -3'.

ChIP-sequencing (ChIP-seq). The ChIP-DNA was amplified to generate a library for sequencing. The workflow consists of fragmentation of whole-genome DNA, end repair to generate blunt ends, A-tailing, adaptor ligation, and PCR amplification. Different adaptors were used for multiplexing samples in one lane. Sequencing was performed on Illumina HiSeq3000 (Illumina, San Diego, CA) for a single read 50 run at the Technology Center for Genomics & Bioinformatics (TCGB) at UCLA. Data quality check was done on Illumina SAV. Demultiplexing was performed with the Illumina Bcl2fastq2 v 2.17 program. Reads were mapped to mouse mm10 genome using the Bowtie1, and MACS2 was used for the peak calling. ChIPseeker was used for the peak annotation. Genome browser representation files were generated by converting ChIP-seq data to bigWig format. This was done using genomeCoverageBed from bedtools v 2.17.0 to generate a bed file, then UCSC bedGraphToBigWig to convert the bed to bigWig format.

Statistical analysis. Data are expressed as mean±SD and analyzed by Permutation *t*-test and Pearson correlation. Per comparison, two-sided *p* values less than 0.05 were considered statistically significant. Multiple group comparisons were made using one-way ANOVA followed by Bonferroni's post hoc test. When groups showed unequal variances, we applied Welch's ANOVA to make various group comparisons. All analyses were used by SAS/STAT software, version 9.4.

Supplementary figures

Fig. S1

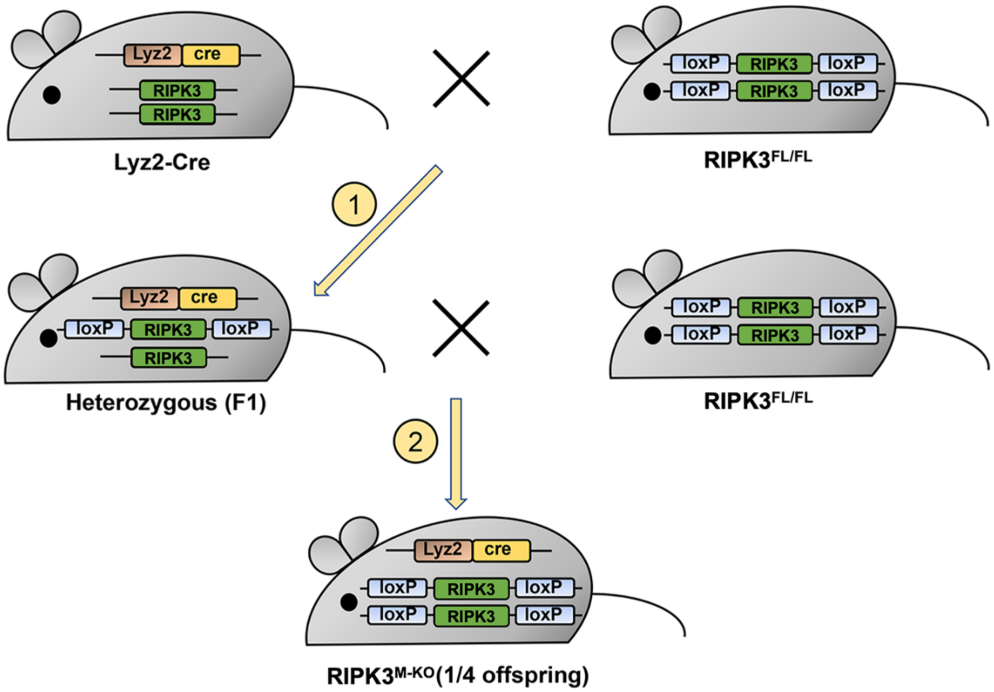

Fig. S1. Schematic illustration of generation of myeloid-specific RIPK3 knockout mice. Two steps were used to generate myeloid-specific RIPK3 KO mice. First, a homozygous loxP-flanked RIPK3 mouse is mated with a homozygous Lyz2-Cre mouse to generate the F1 mice that are heterozygous for a loxP-flanked RIPK3 allele and heterozygous for the Lyz2-cre. Next, these F1 mice were backcrossed to the homozygous loxP-flanked RIPK3 mice, resulting in the generation of myeloid-specific RIPK3 KO mice (RIPK3$^{M\text{-}KO}$, 25% of the offspring), which were homozygous for the loxP-flanked RIPK3 allele and heterozygous for the Lyz2-Cre allele.

Fig. S2

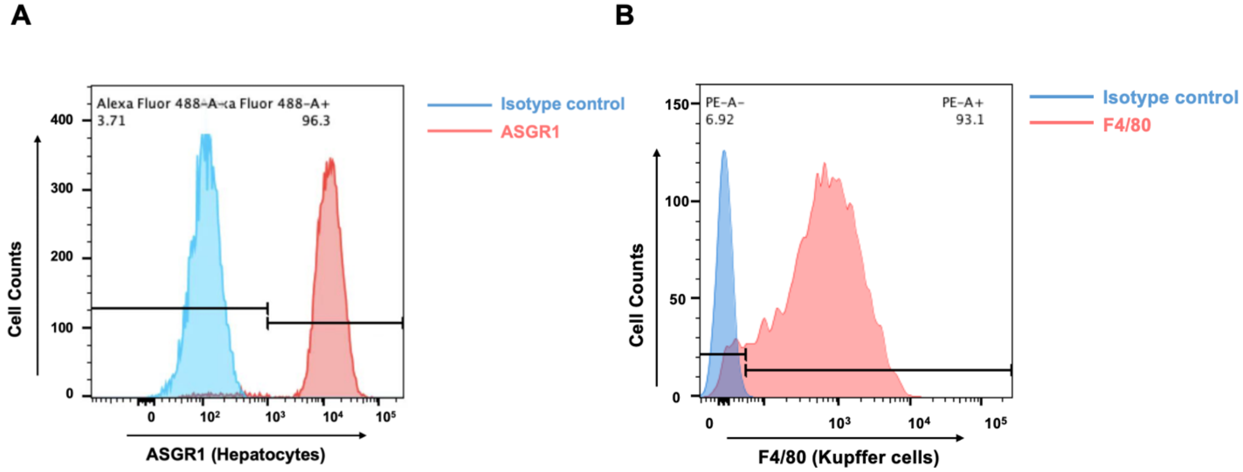

Fig. S2. Purity analysis of isolated hepatocytes and liver macrophages (Kupffer cells) by flow cytometry analysis. (A) The purity of isolated hepatocytes was analyzed by FACS with asialoglycoprotein receptors 1 (ASGR1) antibody (ThermoFisher Scientific, CL488-11739). An isotype control antibody was used (BD Biosciences, 557721). (B) The purity of isolating liver macrophages (Kupffer cells) was analyzed by FACS with F4/80 antibody (BD Biosciences, 565613). Isotype control antibody was used (BD Biosciences, 551436). *Note:* 96.3% of ASGR1+ hepatocytes (Fig. S2A) and 93.1% of F4/80+ macrophages (Fig. S2B) were shown.

Fig. S3

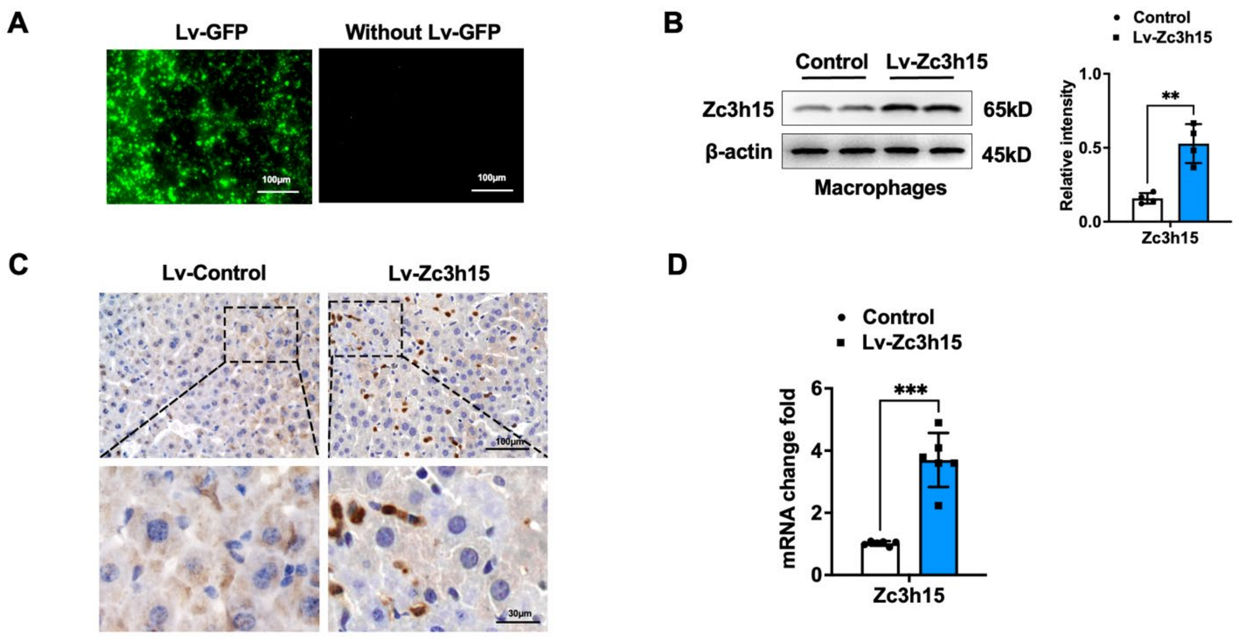

Fig. S3. **Detection of Zc3h15 expression *in vitro* and *in vivo*.** (A) To mimic the transduction efficiency of lentivirus-mediated Zc3h15 (Lv-Zc3h15) in BMMs, Lv-GFP was used to evaluate gene expression in BMMs. *Note:* more than 80% of GFP fluorescence cells were shown in Lv-GFP-transfected BMMs. (B) Western blot analysis and relative density ratio of Zc3h15 protein expression in Lv-Zc3h15-transfected BMMs. (C) Immunohistochemistry staining of Zc3h15 in IR-stressed livers 24h after tail vein injection (n=6 mice/group). Scale bars, 100μm. (D) Quantitative RT-PCR analysis of Zc3h15 mRNA levels in IR-stressed livers 24h after tail vein injection (n=6 samples/group). All Western blots represent four experiments, and the data represent the mean±SD. Statistical analysis was performed using a Permutation t-test. **p<0.01, ***p<0.001.

Fig. S4

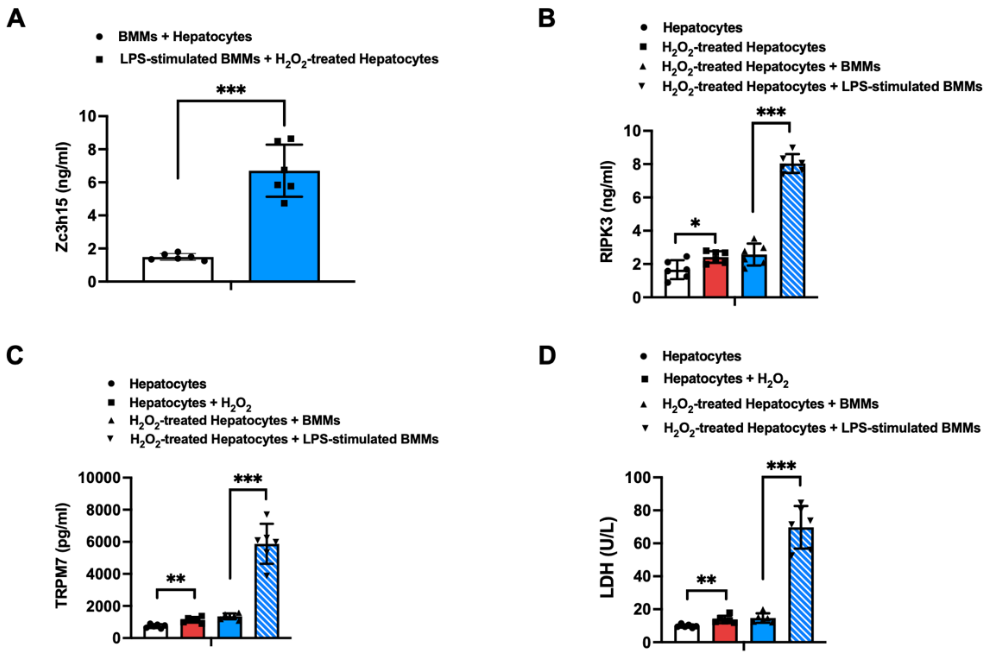

Fig. S4. ELISA analysis of Zc3h15, RIPK3, TRPM7, and LDH assay. Bone marrow-derived macrophages (BMMs) and primary hepatocytes were isolated from the RIPK3$^{FL/FL}$ mice. (A) ELISA analysis of Zc3h15 in cell supernatant after BMM/hepatocyte co-culture. (B-C) ELISA analysis of RIPK3 and TRPM7 in cell supernatant of hepatocytes, H_2O_2-treated hepatocytes alone, H_2O_2-treated hepatocytes plus co-cultured BMM with or without LPS stimulation. (D) LDH assay was performed in the cell culture medium of hepatocytes, H_2O_2-treated hepatocytes alone, H_2O_2-treated hepatocytes plus co-cultured BMM with or without LPS stimulation. The data represent the mean±SD. Statistical analysis was performed using a Permutation t-test. *p<0.05, **p<0.01, ***p<0.001.

Fig. S5

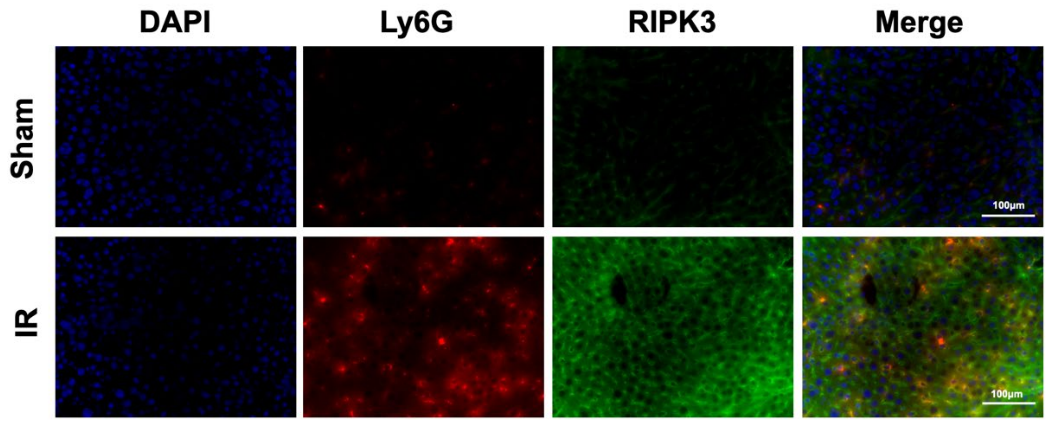

Fig. S5. RIPK3 expression in neutrophils during liver IRI. Immunofluorescence staining of RIPK3 in neutrophils from ischemic livers was performed using RIPK3 and neutrophil marker Ly6G antibodies. *Note:* RIPK3 (green) and Ly6G (red) were shown. DAPI was used to visualize nuclei (blue). Scale bars, 100μm.

Fig. S6

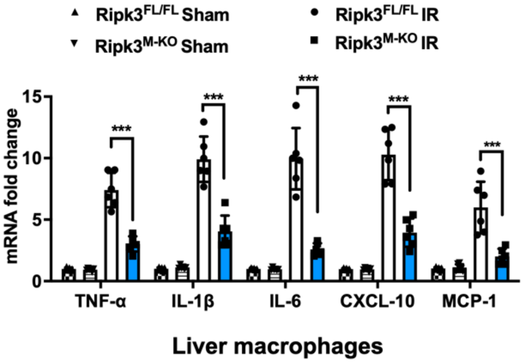

Fig. S6. Proinflammatory cytokine/chemokine expression in isolated liver macrophages. The liver macrophages were isolated from the IR-stressed $RIPK3^{FL/FL}$ and $RIPK3^{M-KO}$ livers. The expression of cytokine/chemokine genes was analyzed by qRT-PCR. *Note:* RIPK3-deficient macrophages ($RIPK3^{M-KO}$) displayed reduced mRNA levels of TNF-α, IL-1β, IL-6, CXCL-10, and MCP-1 compared to the $RIPK3^{FL/FL}$ control cells. The data represent the mean±SD. Statistical analysis was performed using a Permutation t-test. ***$p<0.001$.

Fig. S7

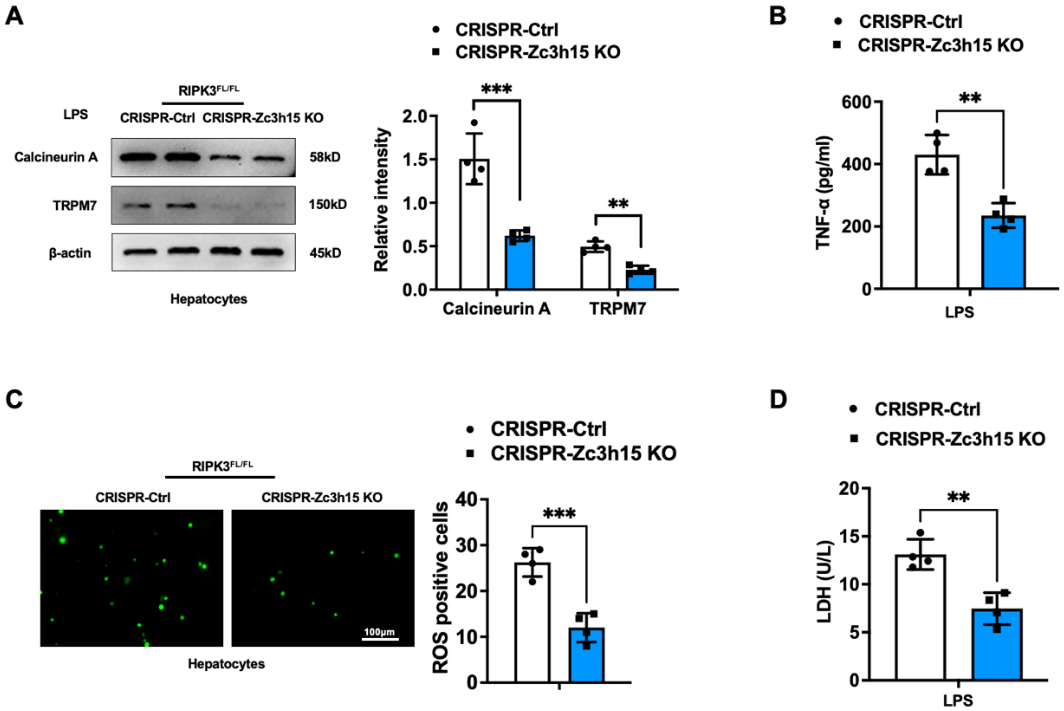

Fig. S7. LPS-stimulated Zc3h15-deficient macrophages reduce RIPK3-induced hepatocyte death after co-culture. (A) BMMs from RIPK3$^{FL/FL}$ mice were transfected with p-CRISPR-Zc3h15 KO or control vector followed by LPS (100 ng/ml) stimulation and then co-cultured with primary hepatocytes without H_2O_2 treatment. Western blot analysis and relative density ratio of Calcineurin A and TRPM7 in hepatocytes. (B) ELISA analysis of TNF-α levels in the co-culture supernatant (n=4 samples/group). (C) Detection of ROS production by Carboxy-H2DFFDA in hepatocytes. Quantification of ROS-producing hepatocytes (green) (n=4 samples/group). Scale bars, 100μm. (D) LDH release in cell medium (n=4 samples/group). All Western blots represent four experiments, and the data represent the mean±SD. Statistical analysis was performed using a Permutation t-test. **p<0.01, ***p<0.001.

Table S1: Primers used in qRT-PCR studies.

Target genes	Forward primers	Reverse primers
HPRT	5'-TCAACGGGGGACATAAAAGT-3'	5'-TGCATTGTTTTACCAGTGTCAA-3'
TNF-α	5'- ACGGCATGGATCTCAAAGAC-3'	5'- AGATAGCAAATCGGCTGACG-3'
IL-6	5'- GCTACCAAACTGGATATAATCAGGA -3'	5'- CCAGGTAGCTATGGTACTCCAGAA -3'
IL-1β	5'-TGTAATGAAAGACGGCACACC-3'	5'-TCTTCTTTGGGTATTGCTTGG-3'
MCP-1	5'-GAAGGAATGGGTCCAGACAT-3'	5'-ACGGGTCAACTTCACATTCA-3'
CXCL-10	5'-GCTGCCGTCATTTTCTGC-3'	5'-TCTCACTGGCCCGTCATC-3'
CXCL-2	5'-CCAACCACCAGGCTACAGG-3'	5'-GCGTCACACTCAAGCTCTG-3'
Zc3h15	5'-TTTGGTCAACAGAATCCACGTC-3'	5'-CAGCAACTACAGGTTTGAACAAC-3'

Supplementary references

[1] Li C, Sheng M, Lin Y, Xu D, Tian Y, Zhan Y, et al. Functional crosstalk between myeloid Foxo1-beta-catenin axis and Hedgehog/Gli1 signaling in oxidative stress response. Cell death and differentiation 2021;28:1705-1719.

[2] Yue S, Zhu J, Zhang M, Li C, Zhou X, Zhou M, et al. The myeloid heat shock transcription factor 1/beta-catenin axis regulates NLR family, pyrin domain-containing 3 inflammasome activation in mouse liver ischemia/reperfusion injury. Hepatology 2016;64:1683-1698.

[3] Suzuki S, Toledo-Pereyra LH, Rodriguez FJ, Cejalvo D. Neutrophil infiltration as an important factor in liver ischemia and reperfusion injury. Modulating effects of FK506 and cyclosporine. Transplantation 1993;55:1265-1272.
